# Genome-wide maps of CPD deamination in yeast reveal the impact of DNA sequence context and nucleosome architecture on cytosine deamination rates

**DOI:** 10.1101/gr.280384.124

**Published:** 2026-01

**Authors:** Marian F. Laughery, Bastian Stark, Benjamin Morledge-Hampton, Steven A. Roberts, John J. Wyrick

**Affiliations:** 1 School of Molecular Biosciences, Washington State University, Pullman, Washington 99164, USA;; 2 Department of Microbiology and Molecular Genetics, University of Vermont Cancer Center, University of Vermont, Burlington, Vermont 05405, USA

## Abstract

UV light induces cyclobutane pyrimidine dimers (CPDs) and other mutagenic lesions in cellular DNA. Cytosine-containing CPDs can subsequently undergo rapid deamination to uracil, a process that has been linked to UV mutagenesis. However, the impact of genomic context and chromatin architecture on CPD deamination rates in cells remains poorly understood. Here, we develop a method known as dCPD-seq to map deaminated CPDs (dCPDs) across the genome of repair-deficient yeast cells at single-nucleotide resolution. Our dCPD-seq data reveal that sequence context significantly modulates CPD deamination rates in UV-irradiated yeast cells, with CPDs in TCG contexts showing particularly rapid deamination rates. Our analysis indicates that rapid CPD deamination can explain why UV-induced mutations are specifically enriched at TCG sequences, both in UV-irradiated yeast cells and in human skin cancers. CPD deamination is suppressed near the transcription start and end sites of yeast genes, which may in part by mediated by DNA-bound transcription factors. Finally, we show that the wrapping of DNA in nucleosomes modulates CPD deamination in yeast cells. Our data indicate that CPD deamination is elevated at minor-in rotational positions where the DNA minor groove faces the histone octamer, likely owing to increased solvent accessibility of the C4 position of the cytosine base. Moreover, we also observe strand-specific enrichment of CPD deamination at rotational positions where the DNA backbone faces out toward the solvent. Taken together, these findings reveal how DNA sequence context and chromatin architecture modulates CPD deamination rates across a eukaryotic genome.

Ultraviolet (UV) light is the primary causative agent of skin cancers because it induces mutagenic damage to DNA (Pfeifer et al. 2005; Brash 2015). The primary class of UV damage is the cyclobutane pyrimidine dimer (CPD), which forms between adjacent pyrimidine bases (i.e., TT, TC, CT, and CC) (Friedberg et al. 2006). Although CPDs form most frequently at TT dinucleotides (Mao et al. 2016), somatic substitutions at TT dinucleotides are relatively infrequent in sequenced skin cancer genomes (Brash 2015; Bonilla et al. 2016; Hayward et al. 2017). Instead, cytosine-to-thymine (C > T) substitutions in dipyrimidine sequences comprise >80% of the mutations observed in skin cancers, including malignant melanoma (Bonilla et al. 2016; Hayward et al. 2017). These observations suggest that cytosine-containing CPDs are especially mutagenic; however, the exact mechanism involved remains unclear.

Although all CPDs are initially formed through the same UV-induced [2+2] cycloaddition reaction, cytosine-containing CPDs are chemically unstable and undergo subsequent spontaneous deamination of the damaged cytosine base to uracil (Setlow et al. 1965; Taylor 2022). Deamination of cytosine bases also occurs in undamaged DNA, but this reaction is very slow, with a half-life of ∼30,000 years in double-stranded DNA and ∼200 years in single-stranded DNA (Frederico et al. 1990). In contrast, the rate of deamination is vastly accelerated for cytosine bases in CPDs, with a half-life of tens to hundreds of hours (Taylor 2022). Because even error-free replicative bypass of a deaminated CPD (dCPD) would result in a C > T substitution, this mechanism could potentially account for the preponderance of C > T substitutions in human skin cancers (Pfeifer et al. 2005; Ikehata and Ono 2011; Jin et al. 2021). The rate-limiting step in the CPD deamination reaction is the hydrolytic attack of a hydroxide or water molecule on the C4 position of the cytosine base (Lemaire and Ruzsicska 1993; Uddin et al. 2014). Extensive biochemical analyses of 5-methylcytosine (5mC)-containing CPDs with variable flanking bases in vitro has revealed that deamination occurs fastest in cytosines with neighboring guanine bases (Cannistraro and Taylor 2009; Taylor 2022). However, to what extent flanking bases affect CPD deamination and subsequent mutagenesis across the genome of UV-irradiated cells is unclear.

Previous biochemical studies have also suggested that CPD deamination can be modulated by DNA-bound proteins, particularly nucleosomes (Cannistraro and Taylor 2010; Song et al. 2011, 2014). Nucleosomes are the fundamental packaging unit of eukaryotic chromatin and are composed of an octamer of histone proteins and ∼147 bp of DNA (Luger et al. 1997). As the nucleosomal DNA wraps around the histone octamer, the minor groove of the DNA backbone is oriented either in toward the histone octamer (minor-in) or away from the histone octamer (minor-out) at 5 bp intervals. We and others have previously shown that both UV-induced CPD formation and somatic mutations in skin cancers are elevated at minor-out and suppressed at minor-in rotational settings (Gale et al. 1987; Mao et al. 2016; Brown et al. 2018; Pich et al. 2018). To what extent the orientation of the DNA minor groove effects CPD deamination is unknown. In between the minor-in and minor-out positions, one DNA strand is oriented with its backbone facing out away from the histone octamer (backbone-out), whereas the complementary backbone faces inward (backbone-in). Previous studies analyzing deamination of a 5mC base in a TCG sequence context found that CPD deamination is significantly elevated at backbone-out orientations in nucleosomes and suppressed at backbone-in orientations in vitro (Song et al. 2011, 2014). However, to what extent nucleosomes modulate CPD deamination in other sequence contexts and across the genome of intact cells remains unclear.

Genome-wide sequencing methods have emerged as powerful tools to understand how different genomic and chromatin contexts impact DNA damage formation and repair (Bohm and Wyrick 2023; Pfeifer and Jin 2024). Recently, Pfeifer and colleagues developed a sequencing method known as circle-damage-seq to measure deamination of CPDs in UV-radiated human cells (Jin et al. 2021). They found that CPD deamination is suppressed near the transcription start site (TSS) and transcription end site (TES) of human genes and that the sequence pattern of CPD deamination shows strong similarity to the COSMIC signature 7 mutation pattern that occurs in melanoma and other skin cancers (Jin et al. 2021). Although these results revealed a promising correlation between deamination and skin cancer mutations, there were some limitations. Specifically, the human cells used in this study were repair-competent, and thus, ongoing CPD repair could complicate analysis of deamination rates, and detection of lesions via cleavage by uracil DNA glycosylase (UDG) precludes the detection of deaminated cytosines in methylated CpG contexts (i.e., TCG and CCG). Moreover, the impact of chromatin organization on deamination rates was largely unexplored, possibly owing to high levels of background deamination during library preparation.

Here, we develop a new method called dCPD sequencing (dCPD-seq) and use it to map dCPDs across the genome of repair-deficient yeast cells in order to characterize the impact of DNA sequence, genomic context, and chromatin organization on CPD deamination.

## Results

**Figure 1.**
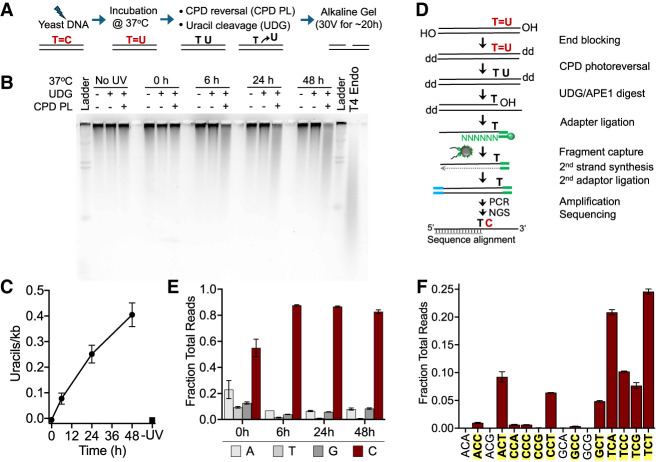
Analysis of deaminated CPDs (dCPDs) in yeast genomic DNA that was UV-irradiated and deaminated in vitro. (*A*) Schematic of the process for quantifying dCPD formation via alkaline gel electrophoresis. Details are available in the Methods. (*B*) Representative SYBR gold–stained alkaline gel showing dCPD levels in UV-irradiated (∼600 J/m^2^ of UVC) yeast genomic DNA at different deamination time points. Control experiments with UDG alone were performed to assess background uracil levels. Irradiated DNA was also independently digested with T4 endonuclease V to confirm UV-induced CPD formation. (*C*) Quantification of alkaline gel electrophoresis results from three independent replicate experiments. Mean ± SEM is depicted. (*D*) Schematic of the dCPD-seq method for detecting deamination of CPDs at single-nucleotide resolution. CPDs are reversed with CPD PL, and genomic uracils are excised with UDG and APE1. Sequencing reads are expected to map adjacent to cytosine bases in dipyrimidine contexts. (*E*) Graph depicting the fraction of total reads mapping immediately upstream of each DNA base in the saccer3 reference genome. dCPD-seq reads map primarily to cytosine bases (red bars) for each deamination time point. Mean and SEM for two independent replicate experiments are depicted for each time point. (*F*) dCPD-seq reads predominantly map to cytosines in dipyrimidine contexts (bold, yellow background). Graph depicts mean and SEM results for two independent replicates of the 24 h time point.

To develop a method to map the deamination of cytosine-containing CPDs, we initially analyzed CPD deamination in yeast genomic DNA that was UV-irradiated in vitro. To quantify the kinetics of CPD deamination in naked DNA in vitro, isolated yeast genomic DNA was UV-irradiated (∼600 J/m^2^ of UVC based on our previous calibration) and subsequently incubated for various times at 37°C to promote deamination (Fig. 1A). This relatively high UV dose was used to increase the number of cytosine-containing CPDs, which comprise only a subset of all CPDs (TT CPDs being the most frequent) (Friedberg et al. 2006). To detect dCPDs, CPDs were repaired using CPD photolyase (CPD PL), and residual uracil lesions resulting from CPD deamination were cleaved with UDG. The resulting cleavage products were analyzed by alkaline gel electrophoresis (Fig. 1B). Although little, if any, DNA cleavage was detectable in the 0 h or no UV samples, we observed increasing levels of DNA cleavage in samples incubated for 6 h, 24 h, and 48 h at 37°C (Fig. 1B). Little if any DNA cleavage by UDG was detected in the absence of CPD PL at these time points (Fig. 1B), indicating that UDG was almost exclusively cleaving uracil lesions present in CPDs. Quantification of these data indicated that uracil formation arising from dCPDs increased at later time points, reaching a frequency of 0.404 ± 0.081 uracil lesions per kilobase of genomic DNA after 48 h incubation at 37°C (Fig. 1C). Parallel experiments using T4 endonuclease V digestion followed by alkaline gel analysis to measure overall CPD induction indicated that this UV dose resulted in 1.76 ± 0.16 CPDs per kilobase in yeast genomic DNA, indicating that ∼23% of all CPDs are deaminated in vitro by 48 h.

To map dCPDs at single-nucleotide resolution across the yeast genome, we developed the dCPD-seq method (Fig. 1D). Yeast genomic DNA (naked DNA) containing dCPDs were blocked on the 3′ end by incubating the DNA with terminal transferase and dideoxyATP or dideoxyGTP (Fig. 1D). The time allotted to this step was relatively short (i.e., 1 h at 37°C) to minimize in vitro deamination of CPDs during library preparation. The resulting DNA fragments were then coincubated with CPD PL and UDG and photo-reactivated with UVA light for 2 h to both reverse the CPD lesions and cleave uracils associated with them. Apurinic/apyrimidinic endonuclease 1 (APE1) was then used to generate a free 3′ hydroxyl (3′OH) immediately upstream of the uracil lesion. Single-stranded DNA with a free 3′OH generated from the UDG and APE1 digestions was ligated to a biotinylated first adapter (Fig. 1D, green), and ligation products were sonicated and purified using streptavidin beads. The complementary strand was synthesized by polymerase extension using a primer complementary to the first adapter, and the other DNA end was ligated to the second adapter (Fig. 1D, blue). Following PCR amplification using primers complementary to both adapters, the resulting libraries were submitted for high-throughput sequencing. The resulting sequencing reads were mapped to the yeast genome, and the location of the uracil lesion was inferred as the DNA base on the opposite strand immediately upstream of the 5′ end of the sequencing read originating from the first adapter.

We sequenced dCPD-seq libraries for two independent deamination time courses, consisting of yeast genomic DNA incubated for 0 h, 6 h, 24 h, and 48 h at 37°C after UVC irradiation. Mapping of the resulting dCPD-seq reads to the yeast genome indicated a significant enrichment of lesions associated with cytosine bases. At later time points (i.e., 6 h, 24 h, and 48 h), >80% of the reads were associated with a cytosine lesion (Fig. 1E). We also observed a weak enrichment of dCPD-seq reads at cytosine bases at the 0 h time point, although their enrichment was not significantly different than that of adenine-associated reads. Moreover, there was relatively little signal associated with 0 h libraries compared with the later time points. Analysis of DNA bases immediately flanking these cytosine lesions indicated that nearly all of these were associated with CPD-forming dipyrimidine sequences (Fig. 1F; Supplemental Fig. S1). Taken together, these findings suggest that the vast majority of dCPD-seq reads are associated with deaminated cytosine bases derived from UV-induced CPD lesions.

### Genome-wide map of CPD deamination in repair-deficient yeast cells

**Figure 2.**
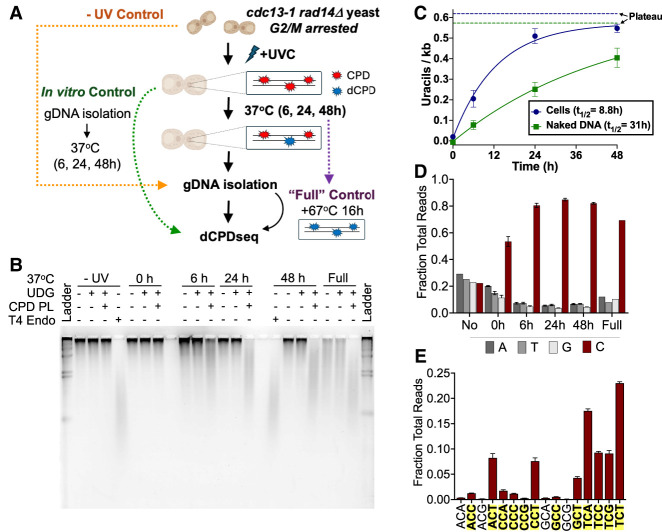
Analysis of dCPD formation in UV-irradiated, NER-deficient cells. (*A*) Schematic of cellular deamination experimental design. Arrested *rad14*Δ *cdc13-1* yeasts were irradiated with ∼600 J/m^2^ UVC and incubated in water at 37°C to maintain arrest and allow deamination to occur. At each time point, cells were harvested and their gDNA extracted for use in library preparation or alkaline gel electrophoresis assay. To create a fully deaminated control, isolated gDNA from the 48 h time point was further incubated for 16 h at 67°C. In vitro control libraries were prepared by isolating genomic DNA from irradiated *rad14*Δ *cdc13-1* yeast and incubating the genomic DNA at 37°C in vitro for the same time points in a thermocycler. (*B*) Representative alkaline gel of CPD deamination experiment performed in cells. (*C*) Quantification of uracils detected via alkaline gel electrophoresis for cellular and in vitro (control) deamination samples. The number of uracils per kilobase represents averages of three independent experiments after subtracting background signal from the UDG-alone digest. Mean ± SEM is depicted. Solid lines indicate the single exponential fit to the cellular and in vitro deamination data. The calculated half-life (*t*_1/2_) from each single exponential fit is listed, and the calculated asymptotic plateau values are indicated as dashed lines. In vitro DNA deamination data are the same as in Figure 1C. (*D*) dCPD-seq libraries prepared from cellular deamination experiments are strongly enriched for reads mapping to cytosine bases (red bars) for each time point. Data depict mean and SEM values from three independent replicates of the 6 h, 24 h, and 48 h time points; two replicates of the 0 h time point; and a single replicate for the no UV and full deamination time points. (*E*) dCPD-seq reads mapping to cytosines occur almost exclusively in dipyrimidine sequence contexts (shown in bold text with yellow highlighting). Graph shows mean and SEM values from three independent replicates of the 24 h time point.

Although our aim was to analyze CPD deamination over a 48 h time course in nucleotide excision repair (NER)–deficient (i.e., *rad14*Δ) yeast cells, these cells are very sensitive to UV irradiation and rapidly die after attempting to replicate their DNA. To overcome this challenge, we arrested cells in the cell cycle prior to UV irradiation using a *cdc13-1* temperature-sensitive strain, which undergoes telomere uncapping and cell cycle arrest in G_2_/M at 37°C (Booth et al. 2001). The *cdc13-1 rad14*Δ strain was grown for 6 h in YPD media at 37°C, resulting in uniform G_2_/M arrest, as determined by microscopy. The arrested cells were then UV-irradiated and subsequently held in water at 37°C for 0 h, 6 h, 24 h, and 48 h (Fig. 2A) prior to cell harvesting and genomic DNA isolation. As a control, we also isolated genomic DNA from yeast immediately after UV irradiation and incubated the resulting genomic DNA at 37°C for the same time points to allow it to deaminate in vitro as naked DNA (i.e., “naked DNA deamination control”) (see Fig. 2A). We also incubated a subset of the isolated 48 h DNA, from either cells or the naked DNA control, an additional 16 h at 67°C (“full” control) (see Fig. 2A). Previous studies have indicated that this treatment results in complete (i.e., “full”) deamination of cytosine-containing CPD lesions (Song et al. 2014).

Alkaline gel analysis of DNA isolated from the cellular deamination time course revealed little if any CPD deamination in the no UV or 0 h samples but increasing levels of deamination at later time points (i.e., 6 h, 24 h, and 48 h) (Fig. 2B; Supplemental Fig. S2). These uracil lesions were specifically associated with CPDs, as little if any UDG cleavage of uracil lesions was detected in the absence of CPD reversal by photolyase (Fig. 2B). To analyze the kinetics of CPD deamination, we fit a single exponential function to the alkaline gel deamination data (Fig. 2C). This yielded a half-life of 8.8 h for deamination of cytosine-containing CPDs in yeast cells. In contrast, similar analysis of CPD deamination kinetics in isolated genomic DNA (i.e., Fig. 1B,C) yielded a half-life of 31 h (Fig. 2C), indicating that CPD deamination occurs more rapidly in intact yeast cells than in solution in vitro. Both fits yielded very similar plateau values for CPD deamination: 0.62 and 0.57 dCPDs per kilobase for cellular and in vitro deamination, respectively, consistent with the fact that the cellular and in vitro samples were irradiated with the same UV dose and therefore should have a similar overall frequency of cytosine-containing CPDs.

Genome-wide analysis of CPD deamination using dCPD-seq in cells at 6, 24, and 48 h after UV irradiation revealed significant enrichment of dCPD-seq reads associated with cytosine bases (Fig. 2D), occurring almost exclusively in CPD-forming dipyrimidine sequence contexts (Fig. 2E; Supplemental Fig. S3). In contrast, there was no enrichment of cytosine-associated dCPD-seq reads in the no UV control and only weak enrichment in the UV-irradiated 0 h sample (Fig. 2D). A similar pattern was observed when dCPD-seq was used to map dCPDs in genomic DNA irradiated in cells but deaminated as naked DNA in vitro (Supplemental Fig. S4, S5).

### CPDs in TCG sequences rapidly deaminate and are associated with elevated UV mutation rates

Because previous biochemical studies have indicated that flanking DNA bases can significantly impact the rate of cytosine deamination in CPD lesions (Tu et al. 1998; Cannistraro and Taylor 2009), we used our dCPD-seq data to characterize the impact of the flanking trinucleotide sequence context on genome-wide CPD deamination rates in intact yeast cells. We quantified the enrichment of dCPD-seq reads at different trinucleotide sequence contexts across the yeast genome by calculating the fraction of dCPD-seq reads in each context relative to the fraction of the genome in that context. This trinucleotide enrichment was plotted for each time point (i.e., 6 h, 24 h, and 48 h) for the cellular deamination time course. As a control, we also plotted the trinucleotide enrichment for the “full” sample, which had undergone complete deamination in vitro, because this should represent the expected enrichment based on UV damage levels alone.

**Figure 3.**
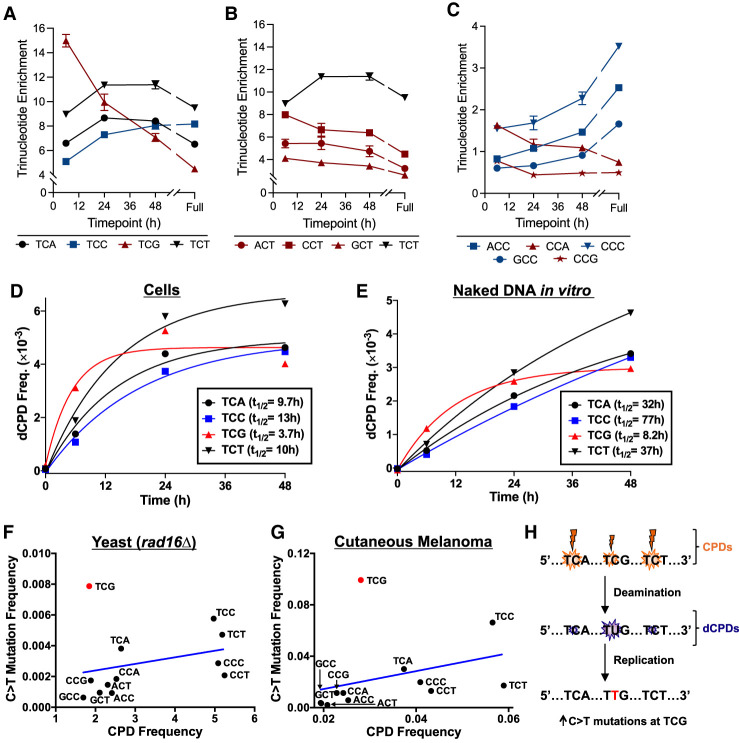
DNA sequence context significantly modulates cellular CPD deamination, likely impacting UV mutagenesis. (*A*–*C*) The fraction of total dCPD-seq reads mapping to each cytosine-central trinucleotide context was normalized by the fraction of the individual trinucleotide sequences in the yeast genome (resulting in “trinucleotide enrichment”) and plotted as a function of 37°C incubation time. Red lines/symbols indicate fast deaminating sequence contexts; blue indicates relatively slow deaminating sequence contexts; and black is intermediate. Results are grouped by TCN (*A*), NCC/CCN (*B*), and NCT contexts (*C*). The 6 h, 24 h, and 48 h results represent the average and SEM from three independent experiments. The 0 h results are from two experiments, and the “full” deamination time point is derived from a single experiment. (*D*) Quantitation of dCPD lesions in cellular deamination samples across the yeast genome at the indicated trinucleotide sequence contexts. dCPD frequency was calculated from frequency of dCPD-seq reads at each trinucleotide context and scaled using the alkaline gel data for the cellular deamination time course. The calculated half-life (*t*_1/2_) of a single exponential fit to each trinucleotide deamination time course is indicated. (*E*) Same as panel *D*, except for UV-irradiated naked yeast genomic DNA that was deaminated in vitro (see Fig. 1). (*F*) The frequency of C > T single-base substitutions in different trinucleotide contexts (e.g., TCG, etc.) is plotted relative to the frequency of UV-induced CPD lesions in each context. Mutation data are derived from whole-genome sequencing of UV-irradiated *rad16*Δ yeast cells (Laughery et al. 2020), whereas CPD frequency is derived from CPD-seq data from UV-irradiated *rad16*Δ yeast cells (0 h time point) (Mao et al. 2020). Blue line indicates the linear regression fit to the data. The mutation frequency at TCG (0.00788; shown in red) is a significant outlier relative to other contexts and lies outside the 95% confidence interval of the predicted value based on its CPD frequency (0.00235 ± 0.00539). (*G*) Same as panel *F*, except mutation frequency is derived from published whole-genome sequencing data from 140 cutaneous melanomas (Hayward et al. 2017), and CPD frequency is derived from published CPD-seq data from UV-irradiated melanoma cells (Lindberg et al. 2019). Again, the mutation frequency at the TCG (0.0993; shown in red) is a significant outlier and lies outside the 95% confidence interval of the value predicted from its CPD frequency (0.0201 ± 0.0683). (*H*) Model depicting potential mechanism for elevated C > T mutations at TCG contexts in yeast and human skin cancers. Although the frequency of CPD lesions is somewhat lower at TCG than other contexts (e.g., TCA or TCT), its deamination occurs much more rapidly, leading to mutation enrichment.

This analysis revealed that different sequence contexts showed distinct deamination profiles (Fig. 3). For example, CPDs in TCG contexts showed approximately 15-fold enrichment after 6 h at 37°C, and this enrichment decreased at later time points, ending at only fourfold enrichment after full deamination (Fig. 3A). This pattern is characteristic of rapidly deaminating CPDs. In contrast, CPDs in a TCC sequence context show the lowest level of enrichment (approximately fivefold) after 6 h at 37°C in cells, and this enrichment increased at later time points (Fig. 3A), a pattern characteristic of slow CPD deamination. Similar analysis of cytosine-containing CPDs in other sequence contexts revealed that NCT sequences (i.e., ACT, CCT, and GCT) showed patterns of trinucleotide enrichment generally consistent with rapid CPD deamination (Fig. 3B), whereas NCC sequences (i.e., ACC, CCC, and GCC) showed patterns consistent with slow CPD deamination (Fig. 3C). We observed similar patterns of trinucleotide enrichment in the in vitro deamination experiments (Supplemental Fig. S6, S7).

To confirm these findings, we used our alkaline gel measurements of the genomic frequency of uracil lesions arising from CPD deamination to normalize the dCPD-seq data and thereby estimate the absolute frequency of dCPDs in each of these trinucleotide sequence contexts across the cellular deamination time course. Analysis of the resulting absolute dCPD frequencies using single exponential kinetics (see Fig. 3D) indicated that CPD deamination in TCG sequences had the shortest half-life (*t*_1/2_ = 3.7 h), whereas TCA and TCT sequence contexts had intermediate half-lives (9.7 h and 10 h, respectively), and TCC sequences had a longer half-life (13 h). Analysis in other contexts confirmed that CPD deamination in NCT trinucleotides showed relatively short half-lives (6 h–10 h) (Supplemental Fig. S8A), whereas NCC sequences showed much longer half-lives (13 h–26 h) (Supplemental Fig. S8B).

A similar analysis in UV-irradiated yeast genomic DNA that was deaminated in solution in vitro showed similar trends. CPDs in TCG contexts had the most rapid deamination rate, with a half-life of 8.2 h (Fig. 3E). In contrast, CPD deamination in other TCN sequence contexts (i.e., TCA, TCC, and TCT) had longer half-lives, ranging from 32 h to 77 h (Fig. 3E). CPD deamination for NCT sequence contexts revealed half-lives ranging from 18 h to 37 h (Supplemental Fig. S8C), whereas in vitro deamination of NCC contexts was too slow to accurately measure the half-life (Supplemental Fig. S8D). The measured deamination half-life of 32 h for CPDs in TCA sequence contexts for yeast genomic DNA deaminated in vitro is roughly consistent with the previously reported half-life of 46 h for CPD deamination in a single ATCA sequence context in vitro (Cannistraro and Taylor 2009). Taken together, this analysis indicates CPDs in a TCG context show much higher rates of cytosine deamination than other sequences, both in cells and in vitro.

We wondered whether rapid deamination of CPDs in TCG sequences might result in a corresponding increase in UV mutagenesis. To test this hypothesis, we analyzed a compendium of UV-induced cytosine-to-thymidine (C > T) mutations identified in a published study using whole-genome sequencing to identify mutations arising in yeast cells repeatedly exposed to UV light (Laughery et al. 2020). We focused specifically on C > T substitutions because they are the mutation signature expected from cytosine deamination to uracil (Pfeifer et al. 2005; Jin et al. 2021). To eliminate potential confounding effects associated with repair of dCPDs, we analyzed UV mutations derived from UV-irradiated *rad16*Δ cells, which are deficient in global genomic NER (Verhage et al. 1994). Because flanking sequence context also modulates the frequency of CPD formation (Lu et al. 2021; Wilson and Wyrick 2024), we compared the frequency of UV-induced C > T substitutions in each trinucleotide sequence context to the frequency of CPD lesions in each sequence context, using our published CPD-seq data (0 h time point) from UV-irradiated *rad16*Δ cells (Mao et al. 2020).

This analysis indicated that trinucleotide sequence contexts with higher levels of initial CPD formation tended to be associated with higher levels of UV-induced C > T substitutions (Fig. 3F). However, the frequency of UV-induced mutations in the TCG sequence context was a significant outlier compared with other trinucleotide sequence contexts, being 3.4-fold more frequent in TCG sequences than expected from their associated frequency of CPDs based on a linear regression model (Fig. 3F). If the TCG sequence context was excluded, then there was a significant positive correlation between CPD levels and UV-induced C > T mutations (r = 0.70, *P* < 0.05), but this correlation was not significant if the TCG context was included (*P* > 0.05).

To further test this hypothesis, we analyzed a compendium of somatic mutations derived from whole-genome sequencing of 140 cutaneous melanomas (Hayward et al. 2017). Unlike in budding yeast, CG dinucleotides in the human genome are frequently methylated, which is known to affect CPD formation when exposed to UVB wavelengths present in sunlight (e.g., Pfeifer et al. 2005; Lindberg et al. 2019; Wilson and Wyrick 2024). To account for this potential confounding factor, we used a published CPD-seq data set derived from melanoma cells that had been irradiated with UVB light (Lindberg et al. 2019), and compared the frequency of CPDs in this data set to the frequency of somatic mutations across different trinucleotide contexts.

This analysis indicated that the TCG sequence context was again a significant outlier to other trinucleotide sequence contexts, with 4.9-fold more mutations than expected based on its CPD levels using the linear regression model (Fig. 3G). There was a significant correlation between CPD levels and somatic mutation frequency across different trinucleotide contexts if the TCG sequence context was excluded (r = 0.72, *P* < 0.05), but this correlation was insignificant if the TCG context was included (*P* > 0.05). These findings indicate that UV-induced mutations in both *rad16*Δ mutant yeast and human melanomas are elevated at fast-deaminating TCG sequences, occurring about three- to fivefold more frequently than expected from the levels of damage formation (Fig. 3H).

### CPD deamination is suppressed near the transcription start and end sites of yeast genes

To determine how CPD deamination is modulated within genes in yeast cells, we analyzed the frequency of dCPDs from our cellular dCPD-seq data between the TSS and TES of about 5000 yeast genes, based on published gene coordinates (Park et al. 2014). Each gene was divided into six equally sized bins, and the number of dCPDs in each bin was divided by the number of cytosines in a CPD-forming dipyrimidine sequence context in each bin to yield the normalized dCPD frequency. Additionally, we also analyzed the normalized dCPD frequency in six flanking bins, each 167 bp in length, three upstream of the TSS and three downstream from the TES.

**Figure 4.**
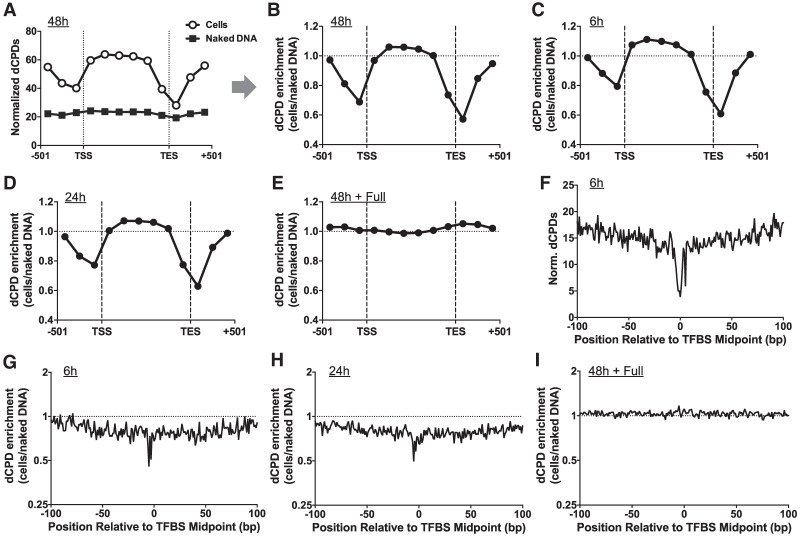
CPD deamination is suppressed near the TSS and TES of yeast genes and adjacent to transcription factor binding sites. (*A*) Normalized frequency of dCPDs following 48 h of deamination after UV irradiation of yeast cells. Lines with open circles and gray squares indicated data for cellular and in vitro (control) experiments, respectively. Data are plotted for about 5000 yeast genes in aggregate. Each gene was divided into six equally sized bins, and the number of dCPD lesions, derived from the count of dCPD-seq reads associated with cytosines in dipyrimidine sequences, was tallied for each bin. dCPDs associated with six 167 bp flanking bins, three upstream of the TSS and three downstream from the TES for each gene, were also tallied. The frequency of dCPD-seq reads in each bin was normalized using the count of cytosine-containing dipyrimidines in each bin. TSS and TES coordinates are from Park et al. (2014). (*B*) Same as panel *A*, except the dCPD enrichment, which is defined as the number of dCPDs in the cellular deamination experiments relative to the in vitro deamination control, is depicted. dCPD enrichment was normalized by the total number of dCPDs in each sample. (*C*–*E*) Same as panel *B*, except for the 6 h (*C*), 24 h (*D*), and full (*E*) deamination samples. (*F*) Normalized dCPD frequency following 6 h deamination in UV-irradiated yeast cells at 1893 binding sites for 78 different yeast transcription factors. The dCPD-seq data at each position were normalized by the frequency of cytosine bases in a dipyrimidine sequence context. TFBS data are from Rossi et al. (2021). (*G*) Same as panel *F*, except plotting dCPD enrichment at yeast TFBSs, which is calculated from the normalized ratio of cellular dCPD-seq reads at each position relative to the in vitro deamination control. (*H*,*I*) Same as panel *G*, except dCPD enrichment for the 24 h (*H*) and full (*I*) deamination sample is plotted.

We initially used this strategy to analyze the 48 h cellular deamination samples. This analysis revealed high levels of dCPDs within genes but lower levels of dCPDs immediately upstream of the TSS and flanking the TES (Fig. 4A). To test whether this was merely a consequence of differences in DNA sequence composition in these genomic regions, we performed similar analysis of the dCPD-seq data derived from 48 h in vitro deamination of naked DNA that had been isolated from UV-irradiated yeast cells (i.e., naked DNA deamination control) (see Fig. 2A). These DNA samples showed nearly constant levels of normalized dCPDs across yeast genes and flanking DNA (Fig. 4A). To quantify the enrichment of CPD deamination in cells, we calculated the ratio of dCPD reads in the cellular samples relative to the naked DNA control at each deamination time point. This ratio was standardized by the overall number of dCPD-seq reads associated with cytosines in dipyrimidine sequences in each sample to yield a dCPD enrichment value (Fig. 4B). This analysis indicated that dCPD enrichment after 48 h of deamination was low (i.e., <1.0) immediately upstream of the TSS and flanking the TES of yeast genes, confirming that CPD deamination is specifically suppressed in regions neighboring the TSS and TES of genes in yeast cells. Similar analysis of dCPD enrichment at the 6 h and 24 h samples again showed suppression of CPD deamination in cells near the TSS and TES of genes (Fig. 4C,D).

To validate these findings, we analyzed the dCPD-seq data for the cellular and naked DNA 48 h samples that had subsequently undergone full deamination in vitro (i.e., 16 h at 67°C). This analysis revealed that the dCPD enrichment for the full deamination samples showed nearly constant deamination across yeast genes and no suppression neighboring the TSS or TES (Fig. 4E). These results confirm that there are similar levels of cytosine-containing CPDs across yeast genes in both the 48 h cellular and in vitro deaminated samples, because complete deamination results in similar levels of dCPDs (i.e., enrichment value of about 1.0).

One possible explanation of this finding is that protein–DNA interactions associated with the TSS and TES of yeast genes may suppress CPD deamination in cells, as suggested by previous studies (Cannistraro and Taylor 2010; Jin et al. 2021; Duan et al. 2024). To test this hypothesis, we used our dCPD-seq data to analyze patterns of CPD deamination at yeast transcription factor binding sites (TFBSs), because these are among the most frequent protein–DNA interactions in regulatory regions like promoters. We used a published yeast ChIP-exo data set that contained 1893 TFBSs derived from 78 different transcription factors (Rossi et al. 2021). Analysis of dCPD-seq data from the 6 h cellular deamination sample indicated that the normalized dCPD levels were decreased at yeast TFBSs (Fig. 4F). To determine if this was because of a decrease in deamination rates at TFBSs in yeast cells, we analyzed the cellular deamination samples relative to the in vitro deamination control. This analysis revealed lower levels of dCPD enrichment near the center of TFBSs in aggregate at the 6 h and 24 h time points (Fig. 4G,H). In contrast, there were no significant difference in dCPD enrichment associated with TFBSs in the fully deaminated control (Fig. 4I). These findings suggest that inhibition of CPD deamination associated with transcription factor binding may contribute to lower levels of dCPDs near the TSS (and potentially TES) of yeast genes.

### CPD deamination is elevated at minor-in rotational settings in yeast nucleosomes

Previous studies have suggested that CPD deamination can be modulated in nucleosomes (Song et al. 2011, 2014), but to what extent this occurs across the genome was previously unclear. To address this question, we used our dCPD-seq data to analyze patterns of CPD deamination in about 10,000 strongly positioned nucleosomes identified in a previously published map of nucleosome positioning in yeast (Brogaard et al. 2012). Analysis of normalized dCPDs in the 6 h cellular deamination sample revealed a roughly periodic pattern of CPD deamination, particularly at distal positions (i.e., −73 to −20 and +20 to +73) in the nucleosome DNA (Supplemental Fig. S9, top). In general, normalized dCPD levels were lower at minor-out rotational settings (Supplemental Fig. S9, dashed lines) and higher at minor-in positions (in between dashed lines). A similar pattern of dCPDs was observed at the 24 h and 48 h cellular deamination samples, although the periodicity was somewhat reduced at the 48 h time point and largely absent from the full deamination control (Supplemental Fig. S9).

**Figure 5.**
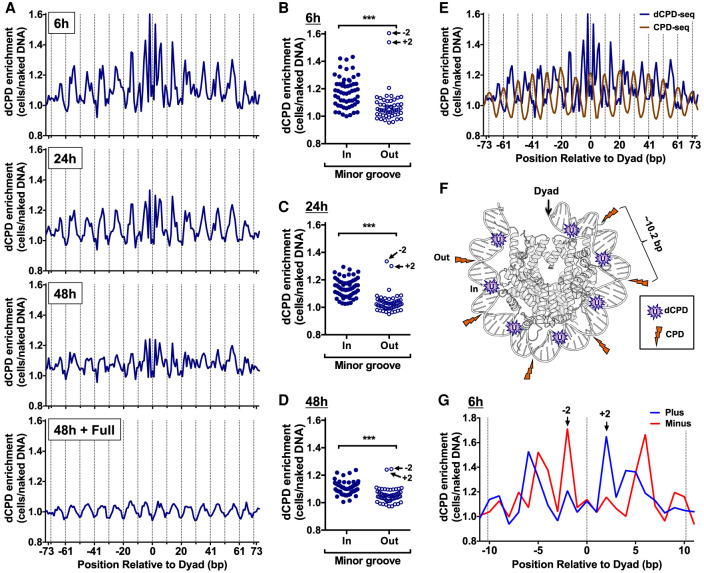
CPD deamination is elevated at minor-in rotational settings and suppressed at minor-out rotational settings in nucleosomes. (*A*) dCPD enrichment in UV-irradiated cells relative to the in vitro deamination control is plotted at each position relative to the central dyad of about 10,000 strongly positioned nucleosomes for the indicated deamination time points. Dashed lines indicate minor-out rotational settings. Nucleosome positioning data are from Brogaard et al. (2012). (*B*–*D*) Plot of dCPD enrichment at minor-in and minor-out rotational settings for 6 h (*B*), 24 h (*C*), and 48 h (*D*) deamination samples. (***) *P* < 0.0001 based on a Mann–Whitney *U* test. (*E*) Comparison of dCPD (6 h time point) and CPD (0 h, immediately following damage) enrichment in strongly positioned yeast nucleosomes. Dashed lines indicate minor-out rotational settings. CPD enrichment was calculated relative to a UV-irradiated naked DNA control and is from Mao et al. (2016). (*F*) Model showing structure of the yeast nucleosome (PDB ID: 1ID3) (White et al. 2001) and highlighting locations of elevated CPD enrichment (orange lightning bolts) and dCPD enrichment (purple U [uracil]). CPD enrichment is elevated at minor-out positions, but CPD deamination is elevated at minor-in positions in nucleosomes. Only one DNA gyre is shown. Image generated using PyMOL. (*G*) Close-up of strand-specific dCPD enrichment in UV-irradiated cells following 6 h deamination relative to the in vitro deamination control. dCPD enrichment is depicted for nucleosome positions within 11 bp of the central dyad of about 10,000 strongly positioned nucleosomes.

To control for the effects of DNA sequence composition in nucleosomal DNA on deamination rates, we also analyzed dCPD enrichment following deamination in yeast cells relative to the in vitro deamination (i.e., naked DNA) control. Importantly, both of these samples were UV-irradiated in cells prior to deamination because nucleosomes affect initial CPD formation (Gale et al. 1987; Mao et al. 2016; Brown et al. 2018). This analysis confirmed that dCPD enrichment in yeast cells relative to the naked DNA control displays a periodic pattern in strongly positioned nucleosomes across the deamination time course (Fig. 5A). Following 6 h deamination, dCPD enrichment was decreased at minor-out rotational settings (Fig. 5A, dotted lines) and elevated at minor-in rotational settings. A similar pattern was observed at the 24 h and 48 h deamination time points, although the magnitude of the differences in dCPD enrichment between the minor-in and minor-out rotational positions was reduced at later time points, including in the full deamination control (Fig. 5A). Statistical analysis revealed significantly higher levels of dCPD enrichment at the minor-in rotational settings relative to the minor-out settings across the deamination time course (Fig. 5B–D).

We also compared dCPD enrichment in strongly positioned nucleosomes with initial CPD formation using our published CPD-seq data (Mao et al. 2016). The results indicated that although CPD and dCPD enrichment both showed clear periodicity in nucleosomes, they had the opposite phasing (Fig. 5E,F). CPD enrichment in UV-irradiated yeast cells relative to the naked DNA control is elevated at minor-out rotational settings (Fig. 5E, dashed lines), whereas dCPD enrichment is suppressed at these same positions. In contrast, at minor-in positions, dCPD enrichment is elevated, whereas CPD enrichment was reduced (Fig. 5E).

**Figure 6.**
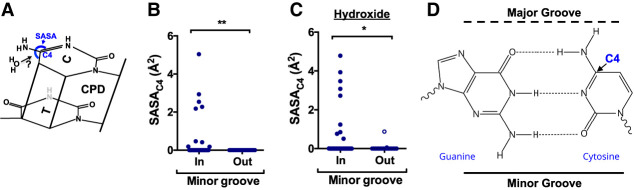
Molecular mechanism underlying elevated CPD deamination at minor-in rotational settings in nucleosomes. (*A*) Chemical structure of a thymine–cytosine CPD. Previous studies indicate that the rate-limiting step of cytosine deamination is hydrolytic attack by a water (H_2_O) molecule on the cytosine C4 position. The accessibility of these C4 position to hydrolytic attack can be quantified using the solvent accessible surface area (SASA). (*B*) Quantification of the SASA_C4_ for cytosine bases in the yeast nucleosome structure (1ID3) for bases at minor-in or minor-out rotational settings. (**) *P* < 0.01 based on a Mann–Whitney *U* test. (*C*) Same as panel *B*, except the SASA_C4_ is calculated for hydroxide (radius ∼ 1.1 Å) instead of water. (*) *P* < 0.05 based on a Mann–Whitney *U* test. (*D*) Chemical structure of the guanine–cytosine base pair, highlighting the fact that the cytosine C4 position is located near the major groove.

We wondered what molecular mechanism was responsible for elevated CPD deamination at minor-in rotational settings in nucleosomes. Previous biophysical and computational studies (Lemaire and Ruzsicska 1993; Uddin et al. 2014) have indicated that the rate-limiting step in the CPD deamination reaction is the hydrolytic attack of a water (or hydroxide) molecule on the C4 position of the cytosine base (Fig. 6A). We reasoned that differences in the solvent accessible surface area (SASA) of these C4 atoms could potentially account for the modulation in CPD deamination. To test this hypothesis, we calculated the SASA of C4 positions (SASA_C4_) to water molecules (radius ∼ 1.4 Å) for all cytosine bases in a published structure of the yeast (*Saccharomyces cerevisiae*) nucleosome (White et al. 2001). Although C4 atoms in most cytosine bases had zero SASA, a subset had a non-zero SASA_C4_ (Supplemental Fig. S10A), indicating that there are significant differences in C4 solvent accessibility depending upon the location of the cytosine base within the nucleosome. We compared the calculated SASA_C4_ between cytosines in minor-out and minor-in rotational settings in the yeast nucleosome structure and found that minor-in rotational settings had significantly higher SASA_C4_ (*P* < 0.01) (Fig. 6B). Multiple cytosines at minor-in rotational settings had nonzero SASA_C4_, whereas none of the cytosines at minor-out positions had nonzero SASA_C4_ (Fig. 6B). The SASA_C4_ of minor-in positions was also significantly elevated relative to minor-out positions in other high-resolution nucleosome structures (Supplemental Fig. S10B,C).

It has also been suggested that in some conditions hydroxide ions may perform the hydrolytic attack on the C4 position of cytosine (Taylor 2022). Hence, we also calculated the SASA_C4_ in nucleosome structures to hydroxide (radius ∼ 1.1 Å) (Supplemental Fig. S10D). Again, we observed significantly higher SASA_C4_ (for hydroxide) at minor-in positions relative to minor-out in the yeast nucleosome structure and other high-resolution nucleosome structures (Fig. 6C; Supplemental Fig. S10E,F). At minor-in rotational settings, the minor groove of the DNA faces toward the histone octamer, whereas the major groove faces the solvent. The C4 position of a cytosine base is located on the major groove side of a G–C base pair (Fig. 6D), which can potentially explain why C4 solvent accessibility is elevated at minor-in rotational settings.

### CPD deamination is elevated at backbone-out positions in nucleosomes

Closer inspection of dCPD enrichment data indicated that although most minor-out positions in strongly positioned nucleosomes showed relatively low levels of dCPD enrichment, two positions immediately flanking the central dyad axis (i.e., −2 and +2) displayed the highest levels of dCPD enrichment (Fig. 5A–D). Analysis of strand-specific dCPD enrichment neighboring the central nucleosome dyad indicated that the peak at the −2 position specifically occurred on the minus strand, whereas the peak at the +2 position specifically occurred on the plus strand (Fig. 5G). Although our previous analysis indicated that CPD deamination is elevated at minor-in positions using the aggregate of both DNA strands, these data suggest that dCPD enrichment is also modulated in a strand-specific manner.

**Figure 7.**
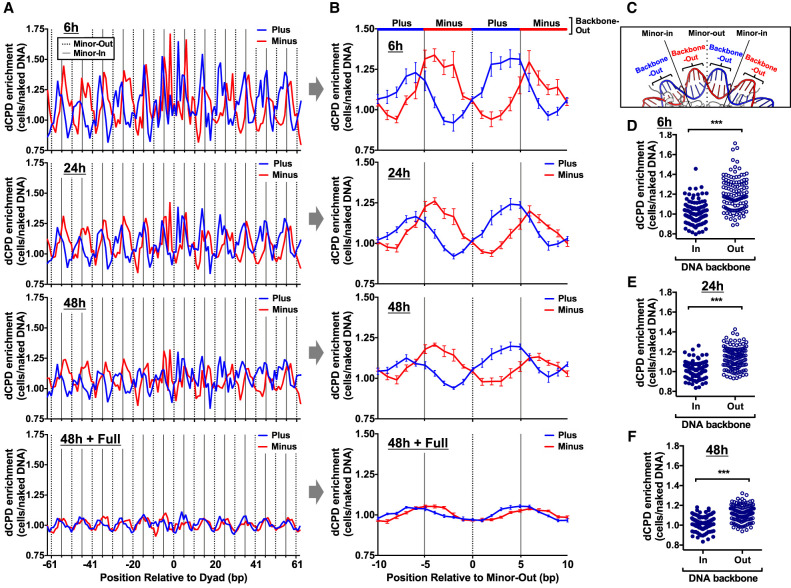
CPD deamination is elevated at DNA backbone-out positions and suppressed at backbone-in positions in nucleosomes. (*A*) Strand-specific dCPD enrichment in UV-irradiated cells relative to the in vitro deamination control is plotted at each position relative to the central dyad of about 10,000 strongly positioned nucleosomes. dCPD enrichment data are plotted for the indicated deamination time points. Dashed lines indicate minor-out rotational settings, and solid lines depict minor-in rotational settings. The mean and SEM depicted for the plus strand is in blue and minus strand in red. Nucleosome positioning data are from Brogaard et al. (2012). (*B*) Same as panel *A*, except showing strand-specific average dCPD enrichment for the 10 bp flanking minor-out rotational settings. Dashed line indicates minor-out, and solid lines indicate flanking minor-in positions. The alternating red and blue colored line at the *top* of the panel indicates which DNA strand is backbone-out (i.e., blue indicates the plus strand is backbone-out, and red indicates the minus strand is backbone-out) at that position in the nucleosome structure. dCPD enrichment is averaged across all minor-out rotational settings in panel *A*. (*C*) Portion of the yeast nucleosome structure (1ID3) highlighting the locations of the minor-in and minor-out rotation settings and DNA backbone-out and backbone-in positions. The plus strand is colored blue and the minus strand red. Image generated using PyMOL. (*D*–*F*) Plot of dCPD enrichment at DNA backbone-in and backbone-out rotational settings in about 10,000 strongly positioned nucleosomes for 6 h (*D*), 24 h (*E*), and 48 h (*F*) deamination samples. (***) *P* < 0.0001 based on a Mann–Whitney *U* test.

To investigate strand-specific patterns of CPD deamination in nucleosomes, we analyzed the dCPD-seq enrichment data for each DNA strand (i.e., plus or minus) separately. This analysis revealed that dCPD enrichment in cells relative to the naked DNA control was elevated near minor-in rotational settings (Fig. 7A, solid lines) in strongly positioned nucleosomes in both DNA strands, consistent with our previous analysis. However, dCPD enrichment for the plus strand was generally elevated immediately to the left of the minor-in position, whereas minus strand enrichment was elevated to the right of the minor-in rotational setting (Fig. 7A). Because the plus and minus DNA strands have an antiparallel orientation, these findings suggest that CPD deamination for each DNA strand is elevated on the 5′ side of the minor-in rotational setting and suppressed on the 3′ side. These strand-specific differences in dCPD enrichment were most pronounced in the 6 h sample, were diminished at 24 h and 48 h, and were essentially absent from the full deaminated control (48 h+Full) (see Fig. 7A).

To quantify these strand-specific differences in CPD deamination, we plotted the average dCPD enrichment values at positions adjacent to each minor-out rotational setting in the nucleosome (Fig. 7B). This analysis confirmed that dCPD enrichment is elevated at minor-in rotational settings (solid lines) and suppressed at minor-out rotational settings (Fig. 7B, dotted lines). It also confirmed strand-specific differences in CPD deamination, as dCPD enrichment for each DNA strand was elevated on the 5′ sides of minor-in rotational settings and reduced on the 3′ side (Fig. 7B).

Closer inspection of the nucleosome structure (Fig. 7C) indicates that between each minor-in and minor-out rotational setting, the sugar–phosphate backbone of one strand faces out toward the solvent (backbone-out), whereas the DNA backbone of the complementary strand is facing in toward the histone octamer (backbone-in). Comparison of these structural features with our dCPD-seq data revealed that dCPD enrichment was specifically elevated at backbone-out orientations (Fig. 7B). Statistical analysis confirmed that strand-specific dCPD enrichment is significantly higher at backbone-out positions relative to backbone-in positions (*P* < 0.0001) (Fig. 7D–F).

**Figure 8.**
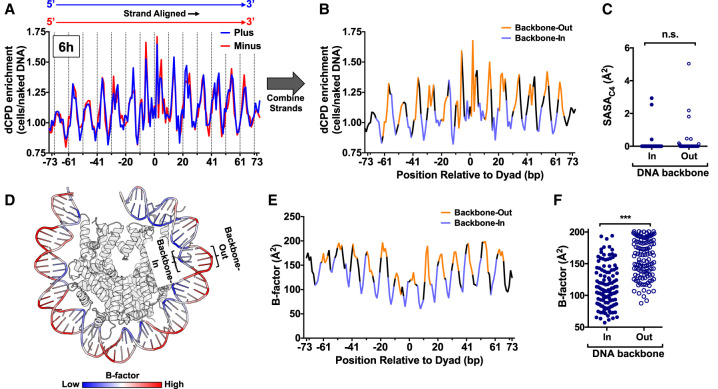
Structural analysis indicates that increased DNA mobility at DNA backbone-out positions promotes CPD deamination. (*A*) Strand-specific dCPD enrichment after 6 h deamination in cells relative to the in vitro control in about 10,000 strongly positioned nucleosomes. Data are the same as Figure 7A (*top* panel), except both DNA strands are aligned in the same 5′–3′ orientation. dCPD enrichment data for the plus strand is in blue and for the minus strand in red. Dashed lines indicate minor-out rotational settings. (*B*) Same as panel *A*, except showing the aggregate dCPD enrichment of both DNA strands each aligned in the 5′–3′ orientation. DNA backbone-out positions are colored orange, and backbone-in positions are in light purple. Intermediate positions are in black. (*C*) Quantification of the C4 solvent accessible surface area (SASA_C4_) for cytosine bases in the yeast nucleosome structure (PDB ID: 1ID3) for bases at minor-in or minor-out rotational settings. (n.s.) Not significant, *P* > 0.05 based on a Mann–Whitney *U* test. (*D*) Visualization of the DNA backbone B-factor for the yeast nucleosome structure (White et al. 2001). Higher B-factors are colored red and lower B-factors in blue. Image generated using PyMOL. (*E*) Quantification of DNA backbone B-factors calculated from the yeast nucleosome structure (PDB ID: 1ID4). Both DNA strands were aligned in the 5′–3′ orientation, and the B-factor values for both strands were averaged. DNA backbone-out positions are colored orange, backbone-in positions are in light purple, and intermediate positions are in black. (*F*) Plot of average DNA backbone B-factors calculated from the yeast nucleosome structure (PDB ID: 1ID4) for DNA backbone-in and backbone-out positions. (***) *P* < 0.0001 based on a Mann–Whitney *U* test.

Analysis of strand-specific dCPD enrichment, in which both the plus and minus DNA strands in strongly positioned nucleosomes were oriented in the same 5′→3′ orientation, revealed very similar patterns of dCPD enrichment between the two strands (Fig. 8A). Combining the dCPD-seq data for the two aligned strands confirmed that dCPD enrichment in cells relative to the naked DNA control was generally elevated at backbone-out positions and suppressed at backbone-in positions (Fig. 8B).

We wondered whether differences in the SASA_C4_ between backbone-out and backbone-in positions of nucleosomal DNA might also explain the observed patterns of dCPD enrichment in nucleosomes. However, our analysis of the yeast nucleosome structure indicated that there was no significant difference in SASA_C4_ between backbone-in and backbone-out positions (*P* > 0.05) (Fig. 8C).

It has been hypothesized that differences in DNA mobility could also promote CPD deamination (Song et al. 2014). To test this hypothesis, we analyzed the B-factor of the DNA backbone for the yeast nucleosome structure, because the B-factor is a measure of DNA mobility (Sun et al. 2019). Inspection of the yeast nucleosome structure, color-coded to visualize the B-factor, indicated that the B-factor was generally low at backbone-in positions and elevated at backbone-out locations (Fig. 8D). Quantification of these data indicated that the DNA backbone B-factor was significantly elevated at backbone-out positions in the nucleosome structure and reduced at backbone-in positions (*P* < 0.0001) (Fig. 8E,F).

## Discussion

The rapid deamination of cytosine bases in CPDs to uracil is thought to play a critical role in promoting C > T mutations in UV-exposed cells and skin cancers (Pfeifer et al. 2005; Ikehata and Ono 2011; Jin et al. 2021). We and others have previously shown that DNA sequence context and packaging of DNA into nucleosomes significantly alters the formation of UV-induced CPDs (Gale et al. 1987; Mao et al. 2016; Brown et al. 2018; Pich et al. 2018; Lu et al. 2021; Wilson and Wyrick 2024), but the impact of these features on CPD deamination across the genome was previously unclear. Here, we use a new method known as dCPD-seq to map genome-wide patterns of CPD deamination. Our data indicate that CPD deamination is significantly modulated by flanking sequence context, occurring most rapidly in TCG sequences in repair-deficient yeast cells and isolated genomic DNA. This propensity for rapid deamination can potentially explain our finding that TCG sequences show a much higher frequency of UV-induced C > T substitutions in both *rad16*Δ yeast and human skin cancers than expected from the frequency of CPDs in this sequence context. Our dCPD-seq data also indicate that nucleosomes significantly modulate CPD deamination rates, as dCPDs are generally elevated at minor-in positions. Structural analysis indicates that these positions are more accessible to hydrolytic attack at the key C4 atom of cytosine bases, suggesting a mechanism for elevated deamination rates at these positions. Our data also revealed strand-specific differences in CPD deamination in nucleosomal DNA, with elevated rates of CPD deamination at backbone-out positions. Structural analysis suggests that such positions have elevated DNA mobility (and flexibility), which has been previously suggested to promote CPD deamination (Song et al. 2014).

Our experimental strategy relies on arresting the UV-irradiated repair-deficient cells by shifting the *cdc13-1 rad14*Δ strain to 37°C, which results in telomere uncapping and resection of a small subset of telomeric regions (Booth et al. 2001). Because single-stranded DNA promotes CPD deamination (Cannistraro and Taylor 2009; Taylor 2022), we tested whether dCPD levels were elevated adjacent to yeast telomeres. We observed a small increase in dCPDs (normalized to cytosine-containing dipyrimidine sequence contexts) within ∼5 kb of telomere ends, particularly in the 48 h time point in the cellular samples, but not in the naked DNA controls (Supplemental Fig. S11A–C), consistent with the hypothesis that CPD deamination is elevated in single-stranded DNA regions induced immediately adjacent to yeast telomeres. These findings also suggest that dCPD-seq can map uracils in single-stranded DNA, consistent with previous reports that CPD PL, UDG, and APE1 can each act on lesions in single-stranded DNA, albeit in some cases with reduced efficiency (Mahaputra Wijaya et al. 2015; Hoitsma et al. 2023). Excluding subtelomeric regions (i.e., regions within 5 kb from a telomere end) did not noticeably impact our analysis of CPD deamination in nucleosomes (Supplemental Fig. S12) or gene coding regions (Supplemental Fig. S13).

Our data indicate that genomic context also regulates CPD deamination, as dCPDs are reduced near the TSSs and TESs of yeast genes. A previous report mapping dCPDs in human cells (Jin et al. 2021) also found decreased CPD deamination near the TSSs and TESs of human genes. This prior report suggested that reduced dCPDs at the TSS could be a consequence of more rapid repair or the impact of DNA-bound transcription factors. Our yeast dCPD-seq data are derived from repair-deficient cells, indicating that differential repair is not responsible for lower levels of dCPDs near the TSSs, at least in yeast. Instead, our analysis suggests that transcription factors and potentially other DNA-bound proteins may play a role in suppressing CPD deamination, consistent with previous reports (Cannistraro and Taylor 2010; Duan et al. 2024). The previous study also suggested that lower levels of dCPDs at the TES might simply be a consequence of differences in sequence composition (i.e., C/G poor sequences) (Jin et al. 2021). However, control dCPD-seq experiments in which the UV-irradiated DNA was deaminated in vitro did not show a decrease in normalized dCPD levels near the TESs of yeast genes, indicating that sequence composition alone is not responsible for this effect. Instead, we propose that DNA-binding by transcription factors or other proteins, such as those involved in transcription termination, suppresses CPD deamination near the TES.

Our data indicate that the DNA sequence immediately flanking a CPD significantly impacts deamination rates, both in vitro and in intact yeast cells. Our data are roughly consistent with previous in vitro studies that characterized CPD deamination rates in specific sequence contexts (Cannistraro and Taylor 2009; Taylor 2022). Consistent with these studies, we observe rapid deamination at TCG sequences, both in yeast cells and in vitro. It has been proposed that the O6 (and possibly N7) functional groups of the neighboring guanine base likely help to promote the hydrolytic reaction of the C4 atom in a 5mC-containing CPD (Cannistraro and Taylor 2009). Our data suggest that a similar mechanism operates for unmethylated cytosines in yeast DNA. Furthermore, analysis of mutations derived from whole-genome sequencing of melanoma tumors or UV-irradiated yeast cells revealed a striking enrichment of C > T substitutions at TCG sequences, despite relatively low damage formation in this same context. This suggests that the presence of a flanking guanine base has opposing effects on mutagenesis, as it suppresses CPD formation by quenching the excited state following UV absorption (Cannistraro and Taylor 2009; Lu et al. 2021) but promotes CPD deamination, which is inherently mutagenic. Although C > T mutations in TCG are the most frequent substitution (after normalizing for the abundance of different sequence contexts) in a data set of 140 cutaneous melanomas (Hayward et al. 2017), this mutation class does not show the same degree of enrichment in the UV-associated COSMIC signatures SBS7a and SBS7b (Forbes et al. 2017). Instead, pyrimidine-associated contexts like TCC have a larger contribution. However, many melanomas and other skin cancers included in the COSMIC database were also determined by nonnegative matrix factorization (NMF) to have enrichment of clock-like SBS1 mutations, which overlaps SBS7a,b primarily at TCG (and CCG) sequence contexts. Hence, it is possible that C > T substitutions in TCG sequence contexts are somewhat unrepresented in SBS7a,b because in some cases they are misassigned to SBS1.

Our dCPD-seq data also indicate that nucleosomes modulate CPD deamination in UV-irradiated cells via two distinct mechanisms. First, dCPDs are enriched at minor-in rotational settings, likely because DNA bending into the minor groove at these positions may facilitate hydrolytic attack of the cytosine C4 position, which at such rotational settings is located in the solvent-facing major groove. We observed residual levels of this pattern even in the full deamination control, potentially reflecting incomplete deamination in nucleosomal DNA or, alternatively, the impact of residual CPD repair by other repair pathways. Notably, CPD formation is elevated at minor-out rotational settings, in which CPD deamination rates are low, and is suppressed at minor-in settings, in which deamination rates are high (Mao et al. 2016). These findings suggest that the same structural features that promote CPD formation may suppress subsequent CPD deamination and vice versa.

Our data indicate that nucleosomes also modulate CPD deamination via a second, strand-specific mechanism. Here, CPD deamination is elevated at backbone-out positions, where the DNA backbone of one strand faces out from the histone octamer toward the solvent, and is suppressed at backbone-in positions. These findings are consistent with previous in vitro studies, which indicated that deamination of a 5mC base in a CPD is stimulated by nucleosomes at backbone-out positions and suppressed at backbone-in positions neighboring the nucleosome dyad (Song et al. 2011, 2014). The magnitude of the difference in dCPD enrichment that we observe in strongly positioned nucleosomes in cells is smaller than that observed for a single artificial nucleosome in vitro, consistent with a previous report (Cannistraro et al. 2015). This discrepancy could be in part because of the proximity of a CPD from the nucleosome dyad. Prior in vitro studies specifically characterized CPDs immediately adjacent to the dyad (Song et al. 2011, 2014), and the biggest effect observed in our cellular studies was similarly at backbone-out positions immediately flanking the dyad (i.e., positions −2 and +2) (see Fig. 5G). This dyad-proximal region is the only segment of the nucleosome in which a single DNA gyre is present (Luger et al. 1997). It is possible that the lack of a neighboring DNA gyre near the dyad might facilitate CPD deamination. It has been previously suggested that enhanced DNA flexibility/mobility at the backbone-out positions in nucleosomes may be responsible for elevated rates of CPD deamination (Song et al. 2014). Consistently, our structural analysis indicates that the B-factor in the yeast nucleosome structure is significantly elevated at backbone-out positions. Taken together, these findings suggest that two distinct biophysical mechanisms promote cytosine deamination in nucleosomes.

Although previous in vitro studies indicated that CPD deamination is elevated at backbone-out positions, these studies did not observe an increase in deamination rates at minor-in rotational settings (Song et al. 2011, 2014). One possible explanation for this discrepancy is that these in vitro studies only analyzed deamination of 5mC in a single sequence context (i.e., TCG). Our dCPD-seq data revealed elevated dCPD enrichment in TCG sequences at backbone-out positions in nucleosomes, consistent with prior in vitro studies (Song et al. 2011, 2014), but no significant enrichment at minor-in positions (Supplemental Fig. S14). One possible explanation is that for TCG sequences, DNA flexibility/mobility may have a dominant effect on deamination rates, as it can regulate the orientation of the CPD relative to the O6 (and possibly N7) functional groups of the neighboring guanine base.

A previous study used comparative genomics and analysis of spontaneous mutations in yeast strains lacking UDG (i.e., *ung1*Δ) to conclude that C > T and other spontaneous mutation classes (i.e., G > T and A > T) are suppressed in nucleosomes, being around twofold lower than in nucleosome-depleted DNA (Chen et al. 2012). Their conclusion was that the packaging of DNA into nucleosomes suppresses spontaneous cytosine deamination. In contrast, analysis of our dCPD-seq data indicate that CPD deamination is enriched in nucleosomes relative to adjacent linker DNA (*P* < 0.01) (Supplemental Fig. S15A,B). This is not simply because of differences in CPD levels, as there is no significant difference between nucleosomes and linker DNA in the fully deaminated samples (Supplemental Fig. S15C,D). These findings indicate that cytosine deamination in CPDs is elevated in yeast nucleosomes and suggest that deamination of CPDs is modulated differently by chromatin than spontaneous cytosine deamination.

In summary, our dCPD-seq data indicate that both sequence context and chromatin architecture significantly modulate CPD deamination rates across a eukaryotic genome. Our data also suggest that rapid deamination of CPDs at TCG sequences results in elevated mutation rates in both UV-irradiated yeast and human melanomas. These findings have potentially important ramifications for understanding mechanisms of UV mutagenesis in skin cancers.

## Methods

### Yeast strains

In vitro UV irradiation and deamination experiments were performed using yeast genomic DNA isolated from strain BY4741. Cellular experiments and naked DNA controls used the NER-deficient (i.e., *rad14*Δ) yeast strain YML461 (Supplemental Table S1), which contains the *cdc13-1* allele (Booth et al. 2001). Details of YML461 construction can be found in the Supplemental Methods.

### Alkaline gel electrophoresis assays of CPD deamination

To quantify the frequency of uracil bases in genomic DNA resulting from CPD deamination, ∼50 µg of DNA based on nanodrop measurements from each of the prepared time points and controls was coincubated with *Escherichia coli* CPD PL and UDG (NEB) in 0.9× CPD PL reaction buffer for 2 h under UVA (∼365 nm) lamps (Spectroline EA-160) at room temperature. Digested samples were analyzed by alkaline gel electrophoresis essentially as previously described (Hodges et al. 2019). A detailed description of UV-irradiated sample preparation and alkaline gel analysis can be found in the Supplemental Methods. Alkaline gel quantifications of dCPDs are reported as the average and SEM resulting from three independent experiments.

### dCPD-seq library construction

The dCPD-seq protocol was adapted from the CPDseq 2.0 method (Duan et al. 2024). Preparation of UV-irradiated genomic DNA and UV-irradiated cellular samples is detailed in the Supplemental Methods. Briefly, ∼160–400 µg of yeast gDNA (measured by NanoDrop spectrophotometry) was incubated with TdT (NEB) and either ddATP or ddGTP to block free 3′OH ends. After phenol:chloroform:isoamyl alcohol extraction and ethanol precipitation, the DNA was resuspended in water and 10× CPD PL buffer (100 mM NaCl, 50 mM Tris HCl at pH 7.5, 20 mM DTT, 1 mM EDTA, 50% glycerol) to a final concentration of 0.7×–1× buffer, and *E. coli* CPD PL and UDG were added to reverse CPDs and excise uracil bases. Reactions were incubated under 365 nm light (Spectroline EA-160) for 2 h at room temperature, and then phenol:chloroform:isoamyl alcohol was extracted and ethanol-precipitated. Because deamination of cytosines in undamaged DNA is dramatically slower than it is in CPDs, performing the photoreversal step early in the dCPD-seq protocol was critical for minimizing unwanted in vitro deamination. APE1 (NEB) was then used to cleave the abasic sugar backbone to yield free 3′OH ends that were ligated to a splint adaptor labeled with biotin on the complementary 5′ end. Ligated DNA was then sonicated (Diagenode Bioruptor 300) to achieve DNA fragments ∼200–400 bp in length, and captured with streptavidin beads (Invitrogen), and washed with 1× saline sodium citrate (SSC) buffer to remove nonspecific binding, and nonbiotinylated strand containing the ligated 3′ adaptor was eluted with 0.15 m NaOH. After synthesis of the complementary strand with EconoTaq (Lucigen) and preparation of DNA ends with NEBNext End Repair and NEBNext dA-Tailing modules, a second adaptor ligation was performed followed by eight to 10 cycles of PCR amplification. Libraries were submitted for either Ion Torrent (WSU LBB1 Core Facility) or Illumina (Azenta) sequencing.

### dCPD-seq data analysis

For analysis of dCPD-seq samples sequenced on the Ion Torrent sequencer, the barcode and 3′ base of the read were removed using custom Perl scripts, as previously described (Mao et al. 2020), and the sequencing reads were aligned to the standard high-quality sacCer3 reference genome using Bowtie 2 (Langmead and Salzberg 2012). The resulting SAM file was converted to a BAM file using SAMtools (Li et al. 2009) and to a BED file using BEDTools (Quinlan and Hall 2010), as previously described (Mao et al. 2016). For dCPD-seq samples sequenced using an Illumina sequencer, adapter trimming sequences were performed by Trimmomatic v0.39 (Bolger et al. 2014), with the parameters “ILLUMINACLIP:[XR-seq_adapter_file]:2:30:10.” Subsequent processing steps were the same as for Ion Torrent sequencing reads. Additional details can be found in the Supplemental Methods.

### Analysis of CPD deamination rates in different sequence contexts

Enrichment of dCPD-seq reads associated with different trinucleotide sequence contexts was calculated from the fraction of total dCPD-seq reads associated with lesions occurring in that particular trinucleotide context (e.g., TCG) divided by the fraction of total trinucleotide sequences in the yeast genome associated with that particular trinucleotide context. This calculation yielded the trinucleotide enrichment score, using the equation for each trinucleotide (e.g., TCG) shown below:

$$\text{TCG trinucleotide enrichment score}=\frac{\left(\text{fraction of dCPD-seqreads in TCG context}\right)}{\left(\text{frac. all trinucleotides in genome in TCG context}\right)}.$$

To calculate the absolute number of dCPDs in each trinucleotide sequence context, we multiplied the trinucleotide enrichment score calculated above by the frequency of uracil lesions in bulk genomic DNA measured by the alkaline gel assay. This yielded an absolute frequency of dCPDs per nucleotide in each trinucleotide context. To calculate the deamination rate for each trinucleotide context, we fit the aggregate dCPD frequency data to a single exponential curve using one-phase association kinetics in the Graphpad Prism software.

### Analysis of structural parameters in nucleosomes

Software for calculating the SASA (*dr_sasa*) was downloaded from GitHub (Ribeiro et al. 2019). The high-resolution X-ray crystal structures of the *Xenopus* (1KX5) and yeast (1ID3) nucleosomes (White et al. 2001; Davey et al. 2002) were obtained from PDB. We analyzed high-resolution structures (3LZ0 and 3LZ1) of the *Xenopus* nucleosome with the strong 601 nucleosomes positioning sequence (Vasudevan et al. 2010). dr-sasa was used to calculate the SASA of all atoms for each structure, either using the default parameter for a spherical solvent (i.e., radius of ∼1.4 Å) to simulate a water molecule or using a custom radius of 1.1 Å to simulate a hydroxide ion. A custom Python script was then used to filter output data for DNA cytosine C4 atoms. Nucleosomal DNA position offsets derived from a previous publication (Stark et al. 2022) were then used to align measurements across each structure, using position 0 as the midpoint/dyad axis of the nucleosomal DNA. For the analysis, data were divided into minor groove in/out positions, as previously described (Stark et al. 2022). Analysis of DNA mobility/flexibility using B-factor values from the yeast nucleosome structure (1ID3) was performed, as previously described, for both DNA strands aligned in the 5′→3′ orientation. B-factor values were averaged for all DNA backbone atoms associated with each nucleotide position and averaged across the two aligned DNA strands.

### Analysis of CPD deamination at yeast genes, TFBSs, and nucleosomes

Analysis of dCPD-seq patterns in yeast genes, TFBSs, and nucleosomes was performed essentially as previously described, using published maps of yeast TSSs and TESs (Park et al. 2014), TFBSs (Rossi et al. 2021), and nucleosome positions (Brogaard et al. 2012). Details can be found in the Supplemental Methods.

## Data access

All raw and processed sequencing data generated in this study have been submitted to the NCBI Gene Expression Omnibus (GEO; https://www.ncbi.nlm.nih.gov/geo/) under accession numbers GSE284099, GSE284778, GSE285152, and GSE285153. Custom scripts are available as Supplemental Code. Source data for the graphs shown in the figures are available at Zenodo (https://zenodo.org/records/17245989) and as Supplemental Data.

## Supplemental Material

Supplement 1

Supplement 2

Supplement 3

Supplement 4

Supplement 5

Supplement 6

Supplement 7

Supplement 8

Supplement 9

Supplement 10

Supplement 11

Supplement 12

Supplement 13

Supplement 14

Supplement 15

Supplement 16

Supplement 17

Supplement 18

Supplement 19
