# Supplementary material for: Genome-wide maps of CPD deamination in yeast reveal the impact of DNA sequence context and nucleosome architecture on cytosine deamination rates
Source: Genome Res. 2026 Jan;36(1):183–96. doi: 10.1101/gr.280384.124 (PMC12887450; doi:10.1101/gr.280384.124)
Supplement: Supplement 1 [file Supplemental_Methods.pdf]

Supplemental Methods

Yeast strain construction

YML461 was constructed in strain YDM14 (LAUGHERY *et al.* 2023) by deleting the *RAD14* gene by homologous recombination-mediated insertion of the KanMX resistance gene. YDM14 is derived from ySR127 (CHAN *et al.* 2015), which contains the *cdc13-1* allele. yML461 (and ySR127) are derived from the CG379 background, which itself was derived from crosses between an S288C strain and A364a strain (MORRISON *et al.* 1991). It is fairly closely related to S288C, having fewer than 1 sequence difference per 1000 base pairs, similar to the commonly used lab strain W303 (ZHUK *et al.* 2023). The *LYS2* gene in this strain background was moved from its endogenous location in chromosome II to a telomere-proximal location in chromosome V, as previously described (YANG *et al.* 2008). This strain was then used to create ySR127, as described (CHAN *et al.* 2015), with a triple mutation reporter (*CAN1-URA3-ADE2*) located in a disrupted *lys2* gene in the chromosome V telomere. This reporter was utilized in other studies, but not in this study.

Preparation of *in vitro* UV-irradiated and deaminated DNA samples for alkaline gel electrophoresis and dCPD-seq library construction

Wild-type genomic DNA was isolated from subcultured BY4741 grown in yeast extract peptone dextrose (YPD) or adenine supplemented YPD (YPDA) media either overnight (for alkaline lysis assays) or to an OD_{600} of 1-2 (dCPD-seq). Genomic DNA was extracted from cells using a method adapted from our previously described protocol (MAO *et al.* 2016). Briefly, harvested cell pellets were resuspended in cell lysis buffer and genomic DNA was then isolated via phenol:chloroform:isoamyl alcohol extraction and bead beating followed by ethanol precipitation. Precipitated DNA was dissolved in 0.1×-1× TE pH 7.5, and digested with 0.15-0.17mg/mL RNase A (ThermoFisher) for 30-40 minutes at 37˚C. Final concentrations were determined using a NanoDrop spectrophotometer.

For *in vitro* UV-irradiation experiments, wild type DNA was diluted to a concentration of 0.8 mg/mL with sterile Millipore water, spotted on glass cover slides, and irradiated on ice to approximately 600 J/m^2 of UVC light, based on our previous calibration. The DNA was then recovered from the slides, pooled, and divided into aliquots which were either stored directly at -20˚C (for the 0h timepoint) or incubated in a thermocycler for 6, 24, or 48 hours at 37˚C. For alkaline gels, 10×TE pH7.5 was added to a final concentration of 0.9-1× TE pH 7.5 prior to incubation. After its incubation period was finished, each sample was stored at -20˚C until all incubations were completed, at which point alkaline gel electrophoresis or sequencing library preparation was performed on all samples concurrently for a given experimental replicate.

Preparation of genomic DNA from cellular and naked DNA deamination experiments for alkaline gel electrophoresis and dCPD-seq library construction

Five milliliters of *rad14*Δ *cdc13-1* yeast (i.e., YML461) were cultured overnight at 23˚C with shaking and added directly to ~30-50mL of prewarmed (i.e. 37˚C) YPDA medium. Subcultures were incubated at 37˚C with shaking for approximately six hours, harvested

by centrifugation, washed with sterile water, and then resuspended to ~40-50 mL with sterile water. The cells were then poured into a tray, exposed to approximately 600 J/m^2 UVC radiation based on our previous calibration, recovered, and then aliquoted into conical tubes and placed in a 37˚C incubator for 6, 24, or 48 hours. During this time, care was taken to avoid exposure of the cells to bright light which could induce photoreactivation of CPDs by the endogenous CPD photolyase. 0h timepoints were not returned to 37˚C; instead, cells were harvested immediately and stored at -80˚C until processing. "No UV" controls were temperature shifted to induce G2/M arrest but were not UV irradiated. gDNA was isolated from cells as described above except RNase A digestion (0.15-0.2mg/mL) was limited to approximately 15 minutes to minimized unwanted *in vitro* CPD deamination. After gDNA isolation, the "Full" deamination control sample was further incubated in a thermocycler set to 67˚C for 16 hours. Samples were stored at -20˚C until used for alkaline gel electrophoresis or dCPD-seq library preparation.

Naked DNA deamination experiments were performed by subculturing, temperature shifting, and irradiating *rad14Δ cdc13-1* yeast as described above. Genomic DNA was directly isolated from these cells as described above and finally resuspended with 10mM Tris, pH 8.0 (replicate 1) or TE pH 7.5 (replicate 2). Replicate 1 was diluted 1:1 with TE 7.5 prior to *in vitro* deamination, whereas replicate 2 was directly deaminated (without further dilution). For deamination experiments, DNA was aliquoted into separate tubes that were incubated in a thermocycler at 37˚C for 6, 24, or 48 hours before storing at -20˚C. 0h timepoints were stored directly at -20˚C. A "Full" deamination control sample was prepared by subjecting the 48h timepoint to an additional 16h incubation at 67˚C.

Alkaline gel electrophoresis assays

To quantify the frequency of uracil bases in genomic DNA resulting from CPD deamination, approximately 50μg of DNA (based on Nanodrop measurements) from each of the prepared timepoints and controls was co-incubated with purified *E. coli* CPD photolyase and UDG (NEB) in 0.9× CPD photolyase reaction buffer (9mM NaCl, 4.5mM Tris-HCl pH 7.5, 1.8mM DTT, 0.09mM EDTA, 4.5% glycerol) for two hours under lamps emitting UVA (~365nm) light (Spectroline EA-160) at room temperature. CPD photolyase enzyme was expressed and purified as previously described (Laughery *et al.* 2020). The amount of photolyase used for experiments was determined by prior titration of the enzyme with irradiated DNA followed by digestion with T4 endonuclease V and gel electrophoresis to assess remaining CPD lesions in genomic DNA. To assess background uracil levels, samples were incubated with UDG alone in either 0.9× photolyase buffer or 1× UDG buffer for 1.75-2 hours. For "No enzyme" samples were buffered with 0.9× CPD photolyase buffer but no enzyme was added to them.

Following enzyme digestion, samples were run on a 1.2% alkaline gel for 19-20 hours at 30V, stained with SYBR Gold (Invitrogen), and imaged on a Typhoon FLA 7000 (GE Healthcare). DNA signal on the gels was quantified using ImageQuantTL and this was used to determine the approximate number of uracils per kilobase using a method adapted from (Bespalov *et al.* 2001). Alkaline gel quantifications are reported as the average and SEM resulting from three independent experiments.

Measuring CPDs using T4 endonuclease V and alkaline gel analysis
To determine the approximate number of lesions produced from our UVC dose, purified genomic DNA was spotted onto glass coverslips at a concentration of ~0.75 ug/uL (stock concentration determined via Nanodrop), placed on a bed of ice, and exposed for 96 seconds (~600 J/m^2 as determined from prior calibration). Spots were then collected, aliquoted into tubes and used for the *in vitro* deamination time course. Aliquots from 48 h deamination samples were then digested with T4 PDG (NEB) in buffer supplied by the manufacturer at 37˚C for approximately two hours prior to alkaline gel analysis alongside other deamination time course samples. Data reported are the average of two independent experimental replicates.

Bioinformatics analysis of dCPD-seq reads
The putative uracil lesion associated with each dCPD-seq read was identified using a custom Perl script as the nucleotide immediately upstream of the 5' end of the mapped dCPD-seq read on the opposite DNA strand. We filtered the resulting damage BED files to only contain dCPD-seq reads associated with cytosine bases in a dipyrimidine sequence context, using BEDTools (QUINLAN AND HALL 2010) and a custom Perl script. The resulting Cdipy files were split into separate files for plus and minus strand lesions and converted to wig files using a custom Perl script. Cytosine bases in dipyrimidine sequence contexts that had no mapped reads were assigned a value of zero reads using a custom Perl script. For subsequent analysis of a dCPD levels associated with yeast genes, transcription factor binding sites, and nucleosomes, only dCPD-seq reads associated with cytosine bases in a dipyrimidine sequence context (i.e., Cdipy files) were analyzed.

Analysis of CPD deamination at yeast genes and transcription factor binding sites
To analyze patterns of CPD deamination in and around yeast genes, we used our published Perl scripts (SELVAM *et al.* 2022) to count the number of dCPD-seq reads associated with bins internal to or flanking each gene. Briefly, each gene was divided into six equally-sized bins encompassing the region between the transcription start site (TSS) and transcription end site (TES; equivalent to the polyadenylation site) of ~5000 yeast genes. TSS and TES data were derived from (PARK *et al.* 2014). Additionally, three flanking bins upstream of the TSS and three flanking bins downstream of the TES were also analyzed. Each of the flanking bins was 167bp in length. The count of dCPD-seq reads in each bin was normalized by the number of cytosine bases in dipyrimidine sequence contexts in each bin. In some cases, we also calculated the enrichment of dCPDs in the cellular samples relative to the *in vitro* deamination control. This enrichment was simply the ratio of dCPD-seq reads in the cellular deamination sample relative to the *in vitro* control, scaled by the total dCPD counts in each sample.
To analyze CPD deamination patterns associated with transcription factor binding sites (TFBS) in aggregate, we used custom Perl scripts to analyze dCPD-seq reads within 100 bp of 1893 binding sites for 78 different yeast transcription factors. The transcription factor binding data were derived from a published ChIP-exo study (ROSSI *et al.* 2021). Again, dCPD-seq read counts at each position were normalized either by the count of

cytosine bases in a dipyrimidine sequence context or by the scaled ratio to the *in vitro* deamination control, as described above.

Analysis of CPD deamination in nucleosomes

Custom Perl scripts were used to analyze dCPD within ~10,000 strongly positioned nucleosomes in yeast. Strongly positioned nucleosomes were defined as having a nucleosome score > 5, using positioning data from (BROGAARD *et al.* 2012). Again, the count of dCPD-seq reads at each position were normalized either by the count of cytosine bases in a dipyrimidine sequence context or by the scale ratio to the *in vitro* deamination control, as described above. Locations of minor-in and minor-out positions in nucleosomes are derived from (CUI AND ZHURKIN 2010).

Supplemental References

Bespalov, V. A., A. Conconi, X. Zhang, D. Fahy and M. J. Smerdon, 2001 Improved method for measuring the ensemble average of strand breaks in genomic DNA. Environ Mol Mutagen 38**:** 166-174.

Brogaard, K., L. Xi, J. P. Wang and J. Widom, 2012 A map of nucleosome positions in yeast at base-pair resolution. Nature 486**:** 496-501.

Chan, K., S. A. Roberts, L. J. Klimczak, J. F. Sterling, N. Saini *et al.*, 2015 An APOBEC3A hypermutation signature is distinguishable from the signature of background mutagenesis by APOBEC3B in human cancers. Nat Genet 47**:** 1067-1072.

Cui, F., and V. B. Zhurkin, 2010 Structure-based analysis of DNA sequence patterns guiding nucleosome positioning in vitro. J Biomol Struct Dyn 27**:** 821-841.

Davey, C. A., D. F. Sargent, K. Luger, A. W. Maeder and T. J. Richmond, 2002 Solvent mediated interactions in the structure of the nucleosome core particle at 1.9 a resolution. J Mol Biol 319**:** 1097-1113.

Laughery, M. F., A. J. Brown, K. A. Bohm, S. Sivapragasam, H. S. Morris *et al.*, 2020 Atypical UV Photoproducts Induce Non-canonical Mutation Classes Associated with Driver Mutations in Melanoma. Cell Rep 33**:** 108401.

Laughery, M. F., D. A. Plummer, H. E. Wilson, B. N. Vandenberg, D. Mitchell *et al.*, 2023 Genome-wide maps of UVA and UVB mutagenesis in yeast reveal distinct causative lesions and mutational strand asymmetries. Genetics 224**:** iyad086.

Mao, P., M. J. Smerdon, S. A. Roberts and J. J. Wyrick, 2016 Chromosomal landscape of UV damage formation and repair at single-nucleotide resolution. Proc Natl Acad Sci U S A 113**:** 9057-9062.

Morrison, A., J. B. Bell, T. A. Kunkel and A. Sugino, 1991 Eukaryotic DNA polymerase amino acid sequence required for 3'----5' exonuclease activity. Proc Natl Acad Sci U S A 88**:** 9473-9477.

Park, D., A. R. Morris, A. Battenhouse and V. R. Iyer, 2014 Simultaneous mapping of transcript ends at single-nucleotide resolution and identification of widespread promoter-associated non-coding RNA governed by TATA elements. Nucleic Acids Res 42**:** 3736-3749.

Quinlan, A. R., and I. M. Hall, 2010 BEDTools: a flexible suite of utilities for comparing genomic features. Bioinformatics 26**:** 841-842.

Rossi, M. J., P. K. Kuntala, W. K. M. Lai, N. Yamada, N. Badjatia *et al.*, 2021 A high-resolution protein architecture of the budding yeast genome. Nature 592**:** 309-314.

Selvam, K., D. A. Plummer, P. Mao and J. J. Wyrick, 2022 Set2 histone methyltransferase regulates transcription coupled-nucleotide excision repair in yeast. PLoS Genet 18**:** e1010085.

Vasudevan, D., E. Y. Chua and C. A. Davey, 2010 Crystal structures of nucleosome core particles containing the '601' strong positioning sequence. J Mol Biol 403**:** 1-10.

White, C. L., R. K. Suto and K. Luger, 2001 Structure of the yeast nucleosome core particle reveals fundamental changes in internucleosome interactions. EMBO J 20**:** 5207-5218.

Yang, Y., J. Sterling, F. Storici, M. A. Resnick and D. A. Gordenin, 2008 Hypermutability of damaged single-strand DNA formed at double-strand breaks and uncapped telomeres in yeast Saccharomyces cerevisiae. PLoS Genet 4**:** e1000264.

Zhuk, A. S., A. G. Lada and Y. I. Pavlov, 2023 Polymorphism of Saccharomyces cerevisiae Strains in DNA Metabolism Genes. Int J Mol Sci 24.
