# Supplementary material for: Genome-wide maps of CPD deamination in yeast reveal the impact of DNA sequence context and nucleosome architecture on cytosine deamination rates
Source: Genome Res. 2026 Jan;36(1):183–96. doi: 10.1101/gr.280384.124 (PMC12887450; doi:10.1101/gr.280384.124)
Supplement: Supplement 2 [file Supplemental_Fig_S1.pdf]

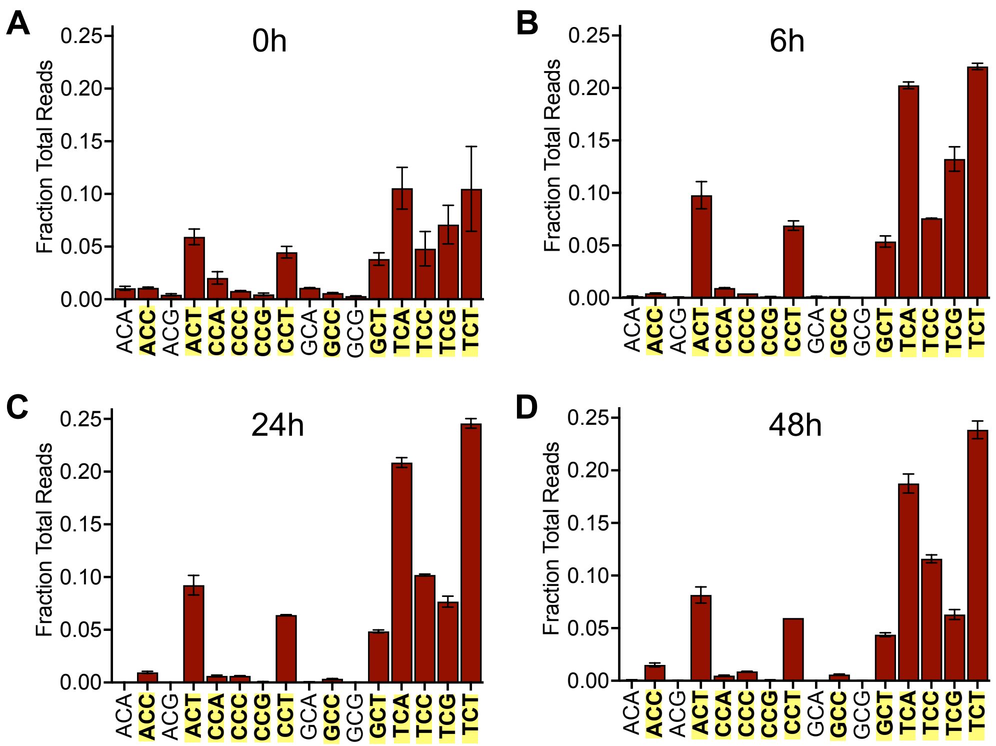

Supplemental Fig. S1. Genome-wide cytosine deamination in CPD lesions is strongly modulated by neighboring bases in isolated yeast genomic DNA incubated at 37˚C for (**A**) 0h, (**B**) 6h, (**C**) 24h and (**D**) 48h following UV irradiation *in vitro*. The fraction of total dCPD-seq reads was analyzed for each cytosine-central trinucleotide. dCPD-seq reads are strongly associated with trinucleotides containing dipyrimidines contexts (shown bolded with yellow highlighting). Panel C is same as Fig. 1F. All deamination timepoints represent the average and SEM from two independent experiments and dCPD-seq library preparations.
