# Supplementary material for: Genome-wide maps of CPD deamination in yeast reveal the impact of DNA sequence context and nucleosome architecture on cytosine deamination rates
Source: Genome Res. 2026 Jan;36(1):183–96. doi: 10.1101/gr.280384.124 (PMC12887450; doi:10.1101/gr.280384.124)
Supplement: Supplement 3 [file Supplemental_Fig_S2.pdf]

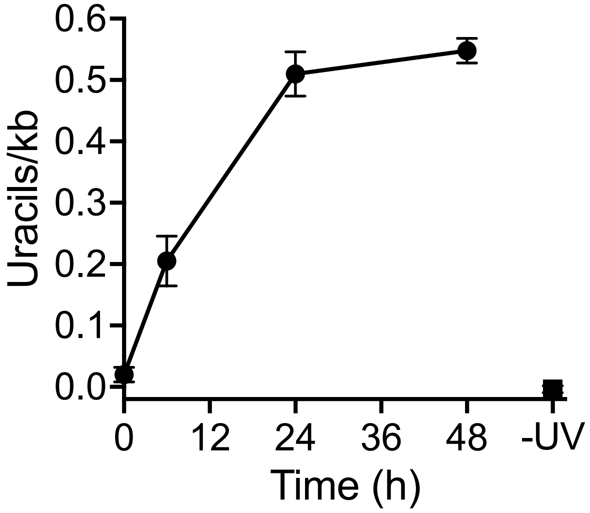

Supplemental Fig. S2. Quantification of uracil formation detected via alkaline gel electrophoresis of CPD deamination experiment performed in cells. The number of uracils per kb represent averages of three independent experiments after subtracting background signal from the UDG-alone digest. Mean ± SEM is depicted.
