# Supplementary material for: Genome-wide maps of CPD deamination in yeast reveal the impact of DNA sequence context and nucleosome architecture on cytosine deamination rates
Source: Genome Res. 2026 Jan;36(1):183–96. doi: 10.1101/gr.280384.124 (PMC12887450; doi:10.1101/gr.280384.124)
Supplement: Supplement 4 [file Supplemental_Fig_S3.pdf]

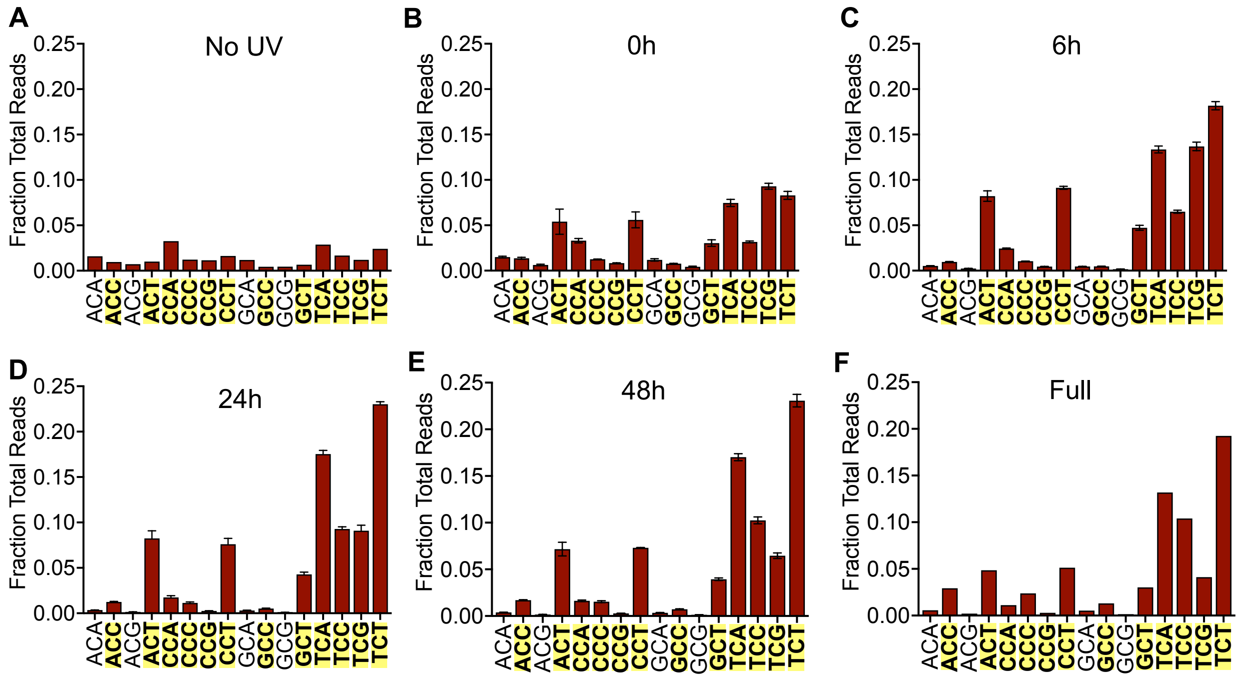

Supplemental Fig. S3. Deamination of cytosine bases in yeast cells is strongly modulated by neighboring bases following UV exposure. **A-E.** Graphs show the fraction of total dCPD-seq reads mapping to each cytosine-central trinucleotide obtained from (**A**) unirradiated cells (No UV, incubated at 37˚C for 48h), or UV-irradiated cells that were subsequently incubated at 37˚C for (**B**) 0h, (**C**) 6h, (**D**) 24h and (**E**) 48h following UV irradiation. A "Full" deaminated control (**F**) was prepared by subjecting irradiated cells that had been incubated for 48h at 37˚C to an additional 16h at 67˚C *in vitro* following gDNA isolation. The fraction of total dCPD-seq reads was analyzed for each cytosine-central trinucleotide sequence. dCPD-seq reads created from UV-irradiated cells strongly correlate to trinucleotides containing dipyrimidines contexts (shown bolded with yellow highlighting). Panel D is same as Fig. 2E. The 6h, 24h, and 48h results depict averages and SEM values from three independent experiments and dCPD-seq library preparations, whereas the 0h timepoint represents the mean and SEM of two

independent experiments. No UV and “Full” deamination timepoints were each derived from a single experiment and library preparation.
