# Supplementary material for: Genome-wide maps of CPD deamination in yeast reveal the impact of DNA sequence context and nucleosome architecture on cytosine deamination rates
Source: Genome Res. 2026 Jan;36(1):183–96. doi: 10.1101/gr.280384.124 (PMC12887450; doi:10.1101/gr.280384.124)
Supplement: Supplement 5 [file Supplemental_Fig_S4.pdf]

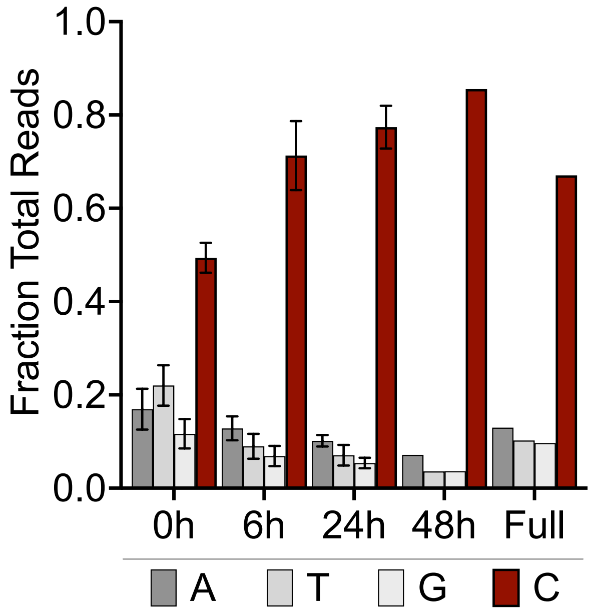

Supplemental Fig. S4. Sequenced libraries generated from *in vitro* deamination of UV irradiated *rad14*Δ *cdc13-1* yeast genomic DNA (naked DNA deamination control) are enriched for reads mapping to cytosine bases in the reference yeast genome. Graph depicts the fraction of total library reads that align immediately upstream of each indicated DNA base for the designated timepoint. 0h, 6h, and 24h results represent the average and SEM from two independent experiments and dCPD-seq library preparations, while 48h and "Full" deamination timepoints were each derived from a single experiment and library preparation.
