# Supplementary material for: Genome-wide maps of CPD deamination in yeast reveal the impact of DNA sequence context and nucleosome architecture on cytosine deamination rates
Source: Genome Res. 2026 Jan;36(1):183–96. doi: 10.1101/gr.280384.124 (PMC12887450; doi:10.1101/gr.280384.124)
Supplement: Supplement 6 [file Supplemental_Fig_S5.pdf]

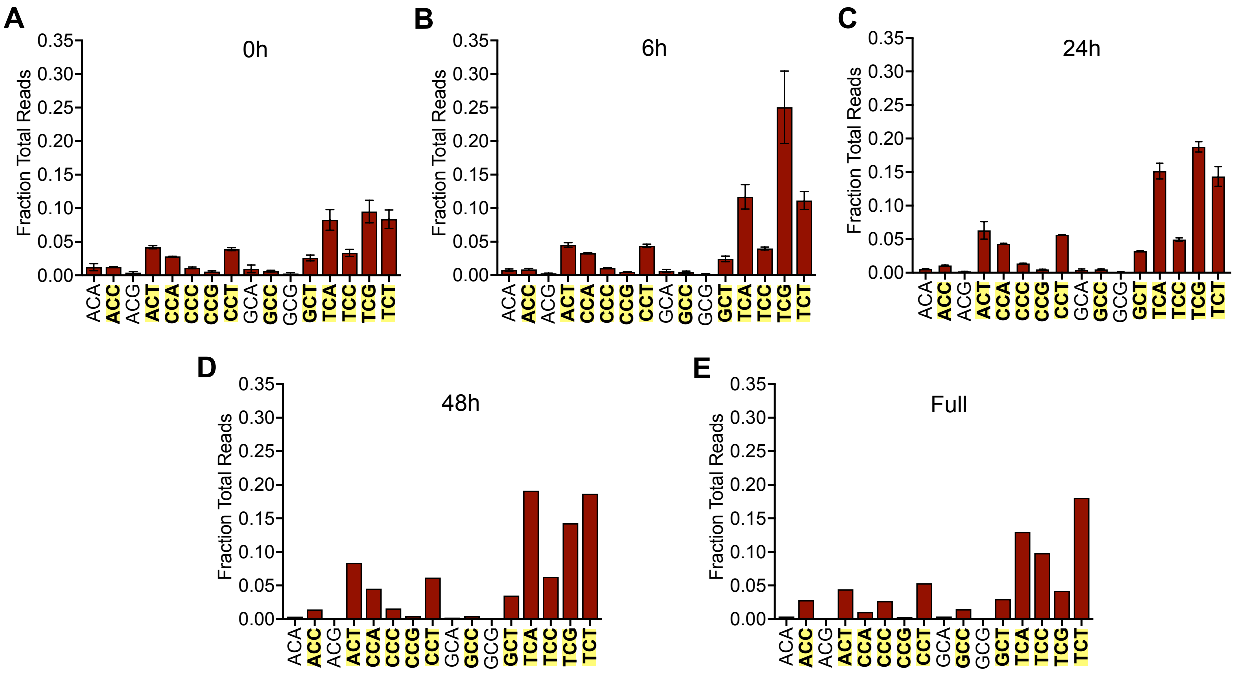

Supplemental Fig. S5. Deamination of cytosine bases in UV-irradiated yeast cells deaminated at 37˚C as naked DNA *in vitro* is strongly modulated by neighboring bases following UV exposure. **A-E**. Graphs show the fraction of total dCPD-seq reads mapping to each cytosine-central trinucleotide obtained from genomic DNA isolated from UV irradiated *rad14*Δ *cdc13-1* G2/M arrested cells that was then incubated as naked DNA *in vitro* at 37˚C for (**A**) 0h, (**B**) 6h, (**C**) 24h, and (**D**) 48h following UV irradiation. Additionally, a (**E**) “Full” deaminated control was prepared by subjecting gDNA that had been incubated 48h at 37˚C to an additional 16h, 67˚C incubation. The fraction of total dCPD-seq reads was analyzed for each cytosine-central trinucleotide. dCPD-seq reads in libraries created from UV-irradiated cells strongly correlate to trinucleotides containing dipyrimidines contexts (shown bolded with yellow highlighting). 0h, 6h, and 24h results represent the average and SEM from two independent experiments and dCPD-seq

library preparations, while 48h and “Full” deamination timepoints are each derived from a single experiment and library preparation.
