# Supplementary material for: Genome-wide maps of CPD deamination in yeast reveal the impact of DNA sequence context and nucleosome architecture on cytosine deamination rates
Source: Genome Res. 2026 Jan;36(1):183–96. doi: 10.1101/gr.280384.124 (PMC12887450; doi:10.1101/gr.280384.124)
Supplement: Supplement 7 [file Supplemental_Fig_S6.pdf]

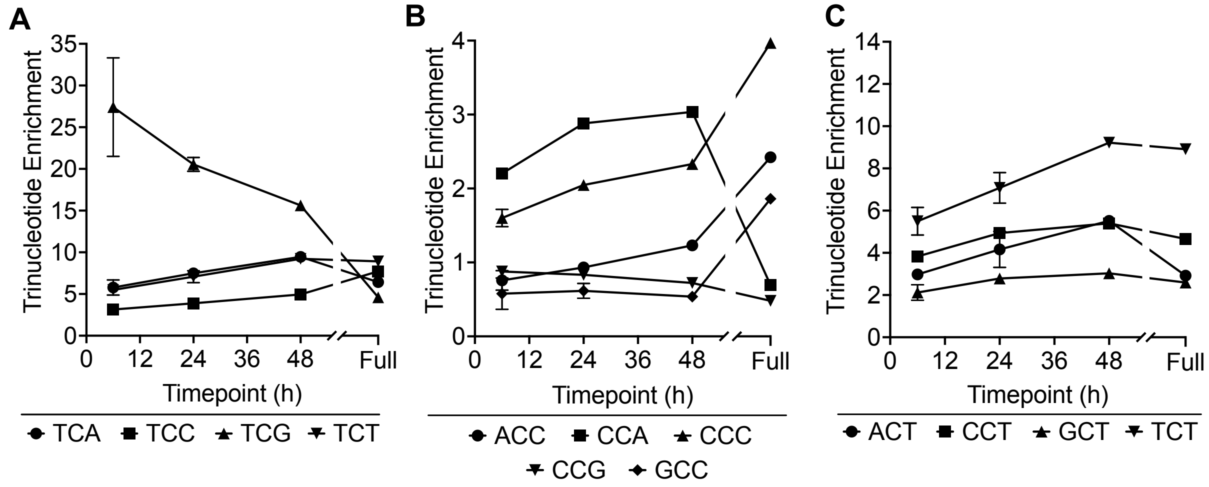

Supplemental Fig. S6. The rate of CPD deamination is influenced by neighboring DNA bases in yeast genomic DNA that was UV-irradiated in cells but deaminated as naked DNA *in vitro*. Isolated genomic DNA from UV-irradiated *rad14*Δ *cdc13-1* yeast cells was incubated at 37˚C *in vitro* following UV exposure and DNA isolation. The fraction of total reads mapping to each cytosine-central trinucleotide context was normalized by the frequency of trinucleotide occurrence (resulting in "trinucleotide enrichment") in the yeast genome and plotted as a function of 37˚C incubation time. "Full" deamination was achieved by subjecting the 48h sample to a second incubation step at 67˚C for 16h. Results are group by (**A**) T**C**N, (**B**) N**C**C/C**C**N, and (**C**) N**C**T contexts. 6h and 24h results represent the average and SEM from two independent experiments and dCPD-seq library preparations, while 48h and "Full" deamination timepoints are each derived from a single experiment and library preparation.
