# Supplementary material for: Genome-wide maps of CPD deamination in yeast reveal the impact of DNA sequence context and nucleosome architecture on cytosine deamination rates
Source: Genome Res. 2026 Jan;36(1):183–96. doi: 10.1101/gr.280384.124 (PMC12887450; doi:10.1101/gr.280384.124)
Supplement: Supplement 8 [file Supplemental_Fig_S7.pdf]

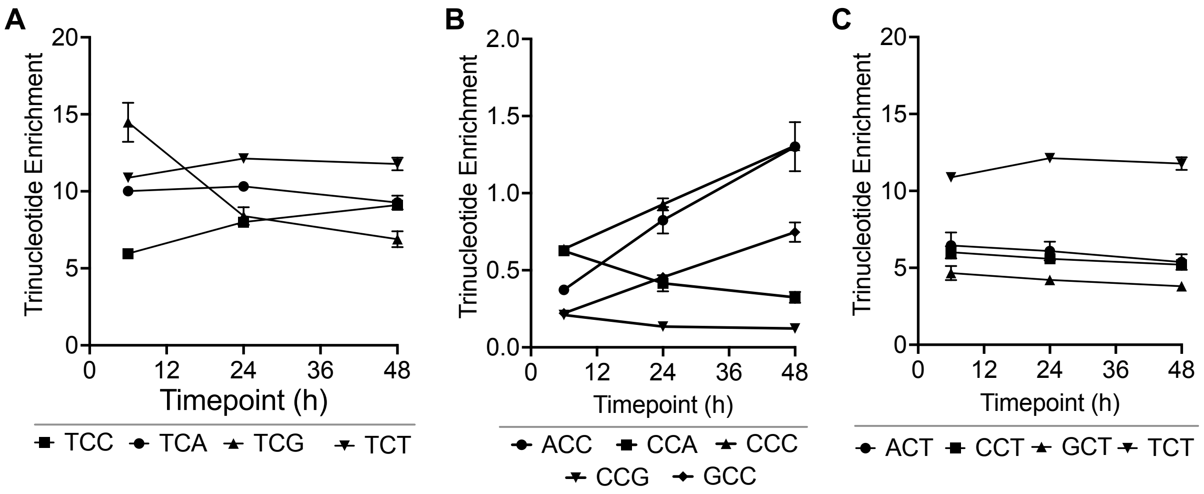

Supplemental Fig. S7. The rate of CPD deamination in isolated yeast genomic DNA *in vitro* is influenced by neighboring DNA bases. The fraction of total reads mapping to each cytosine-central trinucleotide context was normalized by the frequency of trinucleotide occurrence (resulting in “trinucleotide enrichment”) in the yeast genome and plotted as a function of 37˚C incubation time post UV exposure. The results were group by (**A**) T**C**N, (**B**) N**C**C/C**C**N, and (**C**) N**C**T contexts.
