# Supplementary material for: Genome-wide maps of CPD deamination in yeast reveal the impact of DNA sequence context and nucleosome architecture on cytosine deamination rates
Source: Genome Res. 2026 Jan;36(1):183–96. doi: 10.1101/gr.280384.124 (PMC12887450; doi:10.1101/gr.280384.124)
Supplement: Supplement 9 [file Supplemental_Fig_S8.pdf]

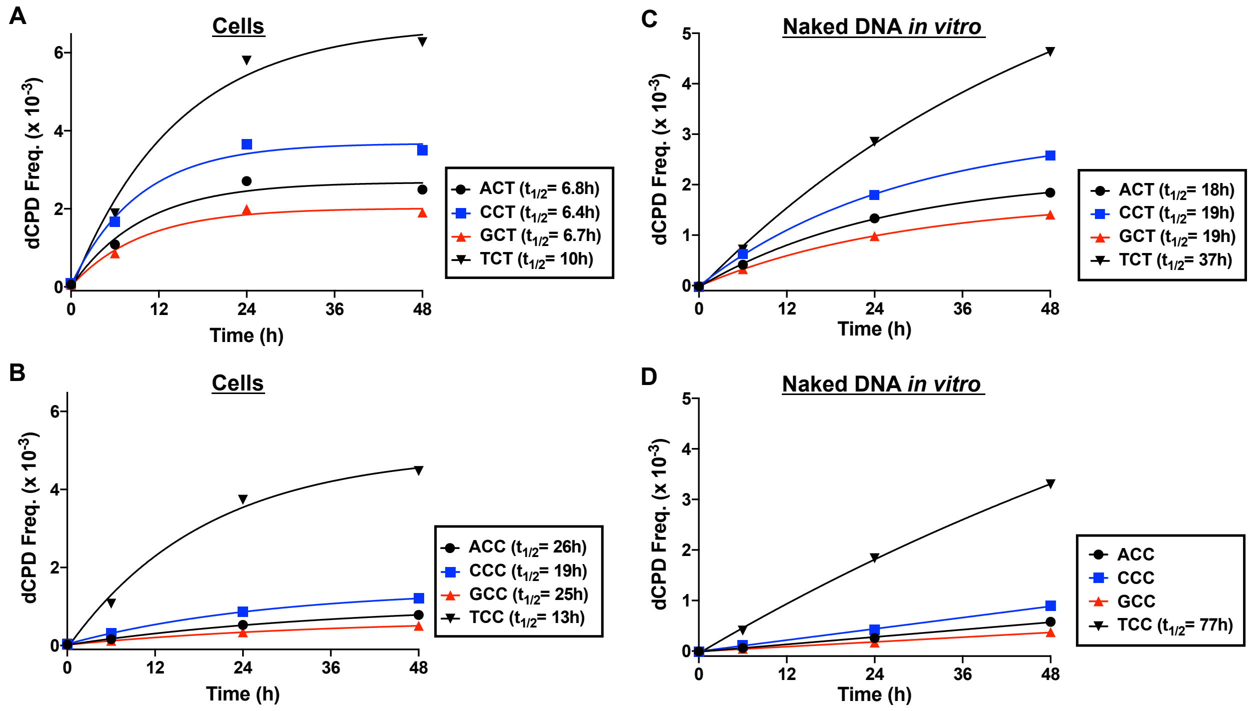

Supplemental Fig. S8. The rate of CPD deamination in NCT and NCC sequence contexts in (**A,B**) UV-irradiated cells or (**C,D**) isolated yeast genomic DNA. **A-B**. Quantitation of dCPD lesions in cellular deamination samples across the yeast genome at the indicated trinucleotide sequence contexts. dCPD frequency (i.e., dCPD frequency per trinucleotide sequence) was calculated from frequency of dCPD-seq reads at each trinucleotide context and scaled using the alkaline gel data for the cellular deamination time course. The calculated half-life ($t_{1/2}$) of a single exponential fit to each trinucleotide deamination time course are indicated. **C-D**, same as panels **A-B**, except for UV-irradiated naked yeast genomic DNA that was deaminated *in vitro*.
