# Supplementary material for: Genome-wide maps of CPD deamination in yeast reveal the impact of DNA sequence context and nucleosome architecture on cytosine deamination rates
Source: Genome Res. 2026 Jan;36(1):183–96. doi: 10.1101/gr.280384.124 (PMC12887450; doi:10.1101/gr.280384.124)
Supplement: Supplement 10 [file Supplemental_Fig_S9.pdf]

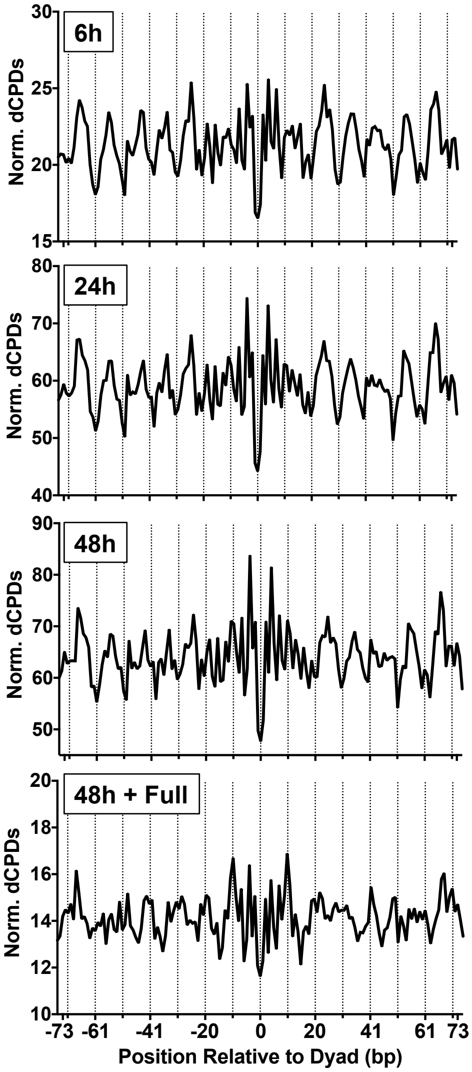

Supplemental Fig. S9. Normalized dCPD-seq reads for the indicated cellular deamination time points are suppressed at minor-out rotational settings and elevated at minor-in rotational settings in strongly positioned nucleosomes. dCPDs were counted at each position relative to the central dyad of ~10,000 strongly positioned nucleosomes. Dashed lines indicate minor-out rotational settings. dCPDs were normalized by the frequency of cytosine bases in dipyrimidine sequences. Nucleosome positioning data is from (BROGAARD *et al.* 2012).
