# Supplementary material for: Genome-wide maps of CPD deamination in yeast reveal the impact of DNA sequence context and nucleosome architecture on cytosine deamination rates
Source: Genome Res. 2026 Jan;36(1):183–96. doi: 10.1101/gr.280384.124 (PMC12887450; doi:10.1101/gr.280384.124)
Supplement: Supplement 11 [file Supplemental_Fig_S10.pdf]

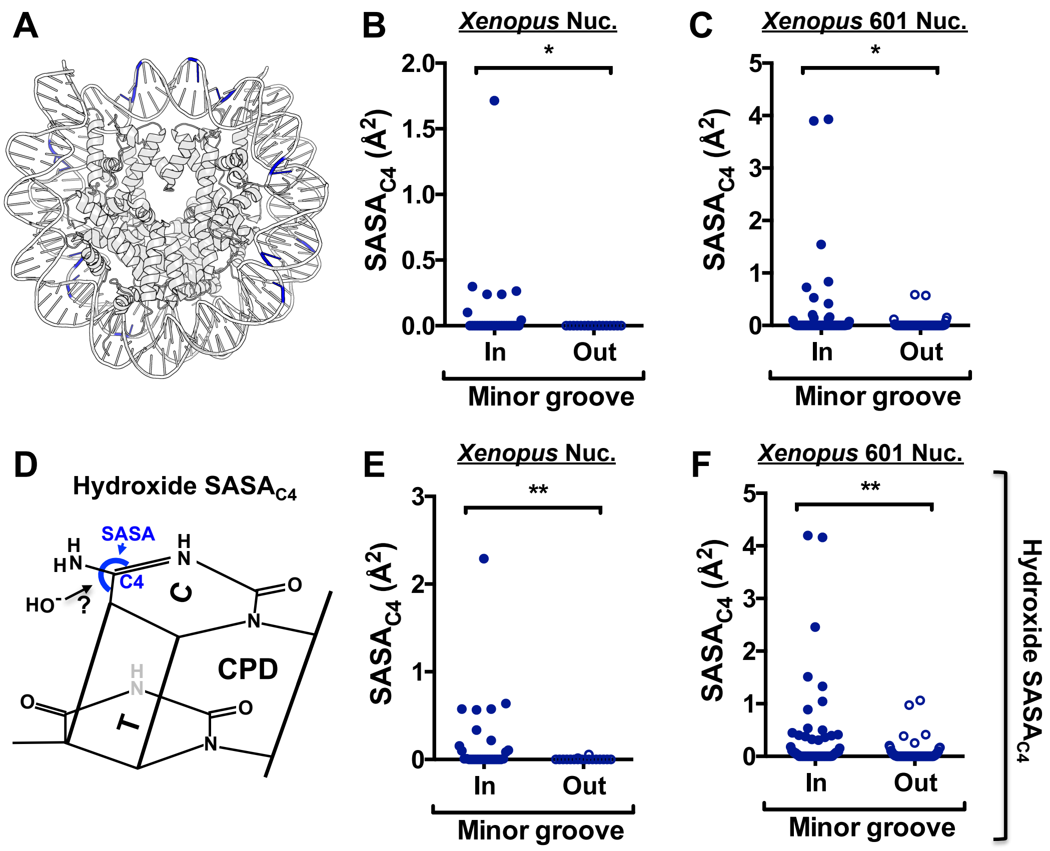


**Supplemental Fig. S10**. Solvent accessible surface area (SASA) of carbon-4 (C4) positions in cytosine bases is significantly elevated at minor-in rotational settings relative to minor-out rotational settings in nucleosome structures. **A.** Visualization of the solvent accessible surface area of the C4 ($SASA_{C4}$) atom for cytosine bases in the yeast nucleosome structure (WHITE *et al.* 2001). Cytosine bases in which the $SASA_{C4}$ is nonzero are highlighted in blue. Image generated using PyMOL. **B,C.** Quantification of the $SASA_{C4}$ for cytosine bases in the *Xenopus* (**B**) 1KX5 nucleosome structure (DAVEY *et al.* 2002) and (**C**) 3LZ0 and 3LZ1 601 DNA sequence-containing nucleosome structures (VASUDEVAN *et al.* 2010). *$P < 0.05$ based on Mann-Whitney *U* test. **D.** Same as Figure 6A, except showing solvent accessible surface area (SASA) for hydrolytic

attack of C4 atom in cytosine by hydroxide. **E,F.** Quantification of the $SASA_{C4}$ for hydroxide (radius ~1.1Å) for cytosine bases in the *Xenopus* (**E**) 1KX5 nucleosome structure (DAVEY *et al.* 2002) and (**F**) 3LZ0 and 3LZ1 601 DNA sequence-containing nucleosome structures (VASUDEVAN *et al.* 2010). **P < 0.01 based on Mann-Whitney *U* test.
