# Supplementary material for: Genome-wide maps of CPD deamination in yeast reveal the impact of DNA sequence context and nucleosome architecture on cytosine deamination rates
Source: Genome Res. 2026 Jan;36(1):183–96. doi: 10.1101/gr.280384.124 (PMC12887450; doi:10.1101/gr.280384.124)
Supplement: Supplement 12 [file Supplemental_Fig_S11.pdf]

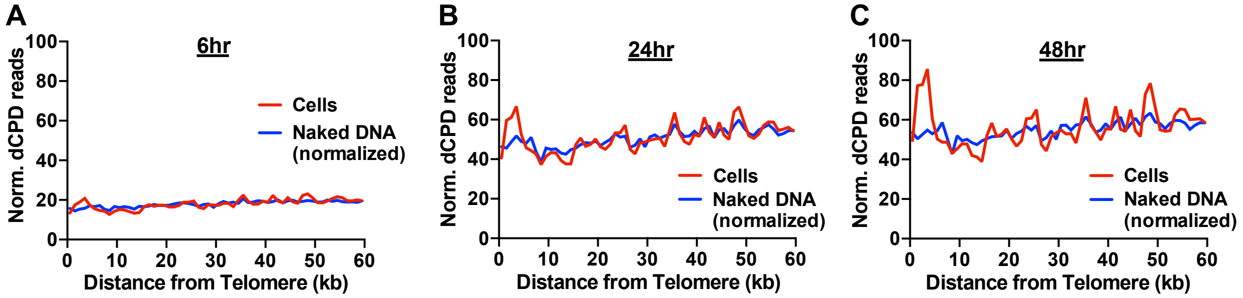

Supplemental Fig. S11. Slight increase in deaminated CPDs (dCPDs) in telomere-proximal regions (i.e., within ~5 kb from telomere end) in cells deaminated for (**A**) 6h, (**B**) 24h, and (**C**) 48h, but not the naked DNA deamination control. dCPD-seq reads were aggregated in 1kb bins relative to the nearest telomere and were normalized to the number of cytosines in a dipyrimidine context in each bin. Naked DNA deamination control was normalized/scaled to match the cellular samples.
