# Supplementary material for: Genome-wide maps of CPD deamination in yeast reveal the impact of DNA sequence context and nucleosome architecture on cytosine deamination rates
Source: Genome Res. 2026 Jan;36(1):183–96. doi: 10.1101/gr.280384.124 (PMC12887450; doi:10.1101/gr.280384.124)
Supplement: Supplement 13 [file Supplemental_Fig_S12.pdf]

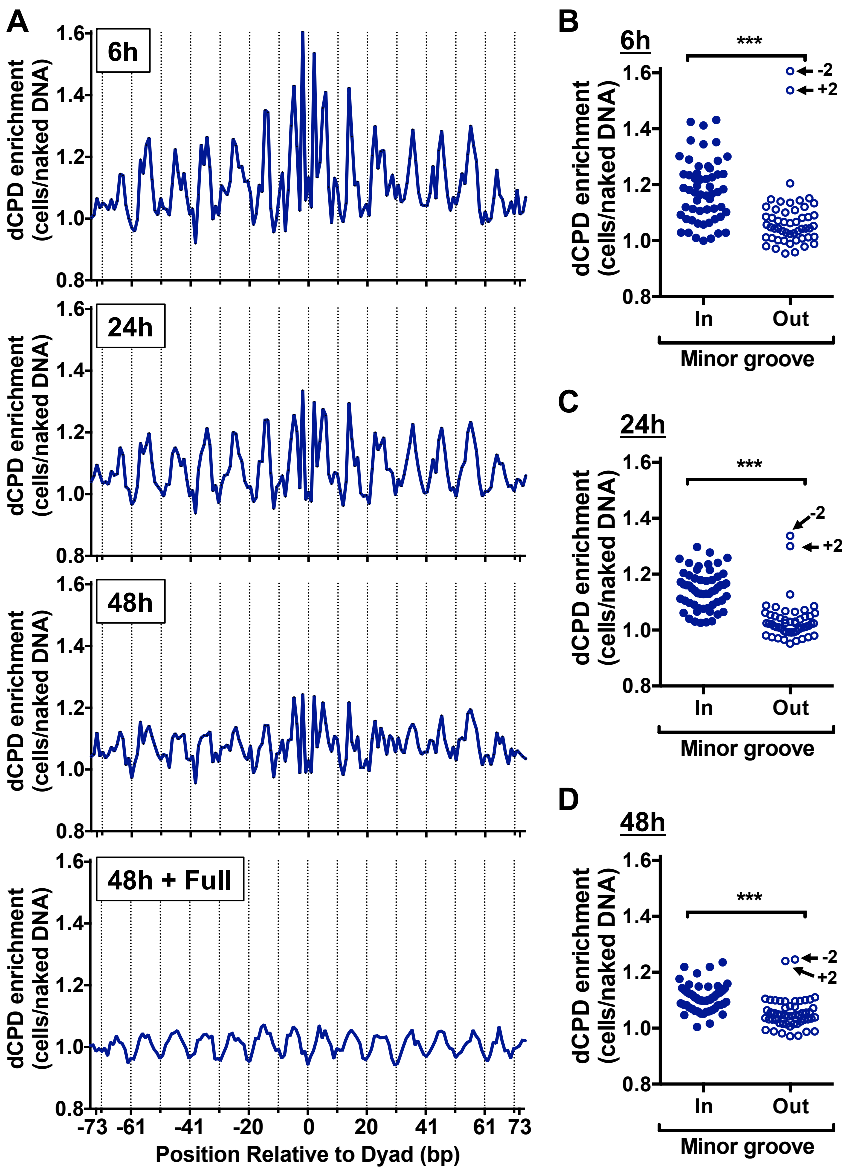

Supplemental Fig. S12. Same as Fig. 5A-D, except nucleosome dyads located within 5 kb of telomere end were excluded from the analysis.
