# Supplementary material for: Genome-wide maps of CPD deamination in yeast reveal the impact of DNA sequence context and nucleosome architecture on cytosine deamination rates
Source: Genome Res. 2026 Jan;36(1):183–96. doi: 10.1101/gr.280384.124 (PMC12887450; doi:10.1101/gr.280384.124)
Supplement: Supplement 14 [file Supplemental_Fig_S13.pdf]

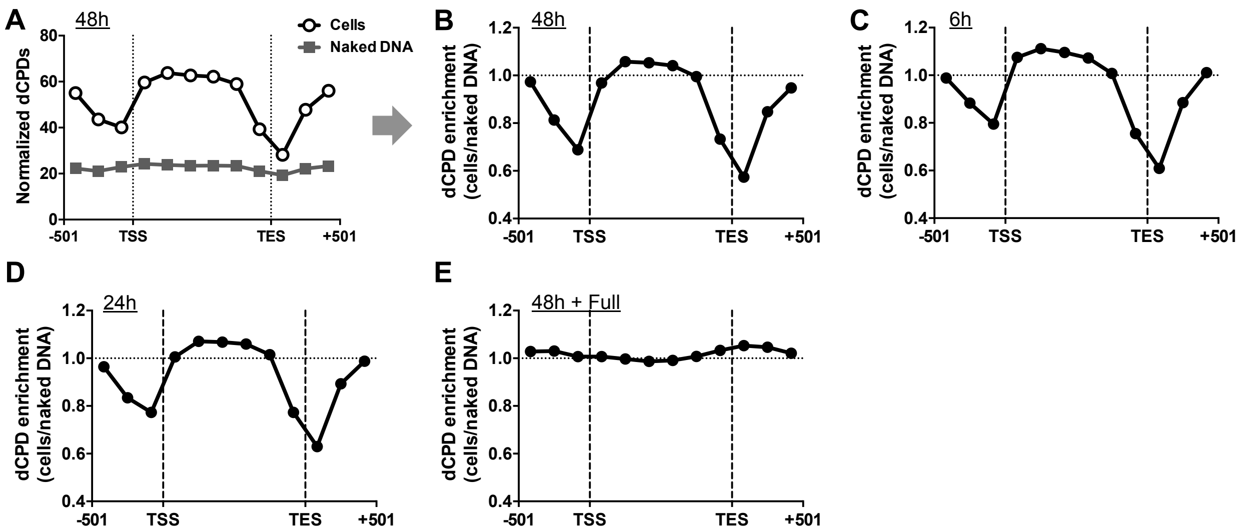

Supplemental Fig. S13. Same as Fig. 4A-E, except genes in which the TSS and/or TES were located within 5 kb of telomere end were excluded from the analysis. The *CAN1, URA3, ADE2,* and *LYS2* genes, which are located in the telomere-proximal mutation reporter, were also excluded.
