# Supplementary material for: Genome-wide maps of CPD deamination in yeast reveal the impact of DNA sequence context and nucleosome architecture on cytosine deamination rates
Source: Genome Res. 2026 Jan;36(1):183–96. doi: 10.1101/gr.280384.124 (PMC12887450; doi:10.1101/gr.280384.124)
Supplement: Supplement 15 [file Supplemental_Fig_S14.pdf]

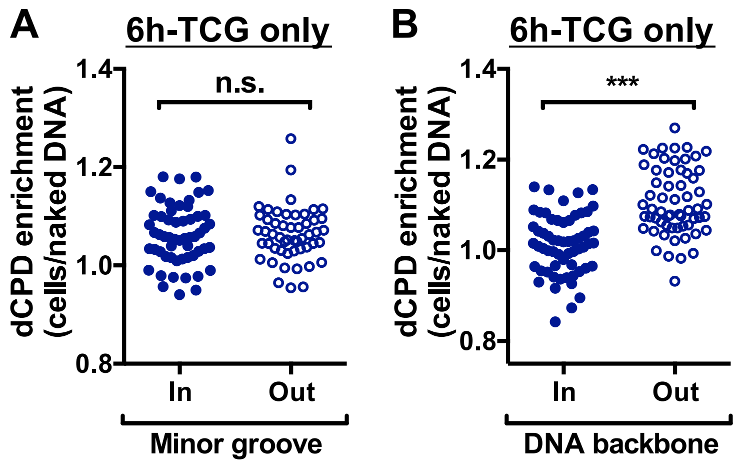

Supplemental Fig. S14. Deaminated CPDs (dCPDs) enrichment in cells versus the naked DNA deamination control for TCG sequence contexts are not significantly elevated in (**A**) minor-in versus minor-out rotational settings, but are significantly elevated in (**B**) DNA backbone-out positions relative to backbone-in positions. ***P < 0.0001; n.s. (not significant) indicates P > 0.05, based on Mann Whitney *U* test.
