# Supplementary material for: Genome-wide maps of CPD deamination in yeast reveal the impact of DNA sequence context and nucleosome architecture on cytosine deamination rates
Source: Genome Res. 2026 Jan;36(1):183–96. doi: 10.1101/gr.280384.124 (PMC12887450; doi:10.1101/gr.280384.124)
Supplement: Supplement 16 [file Supplemental_Fig_S15.pdf]

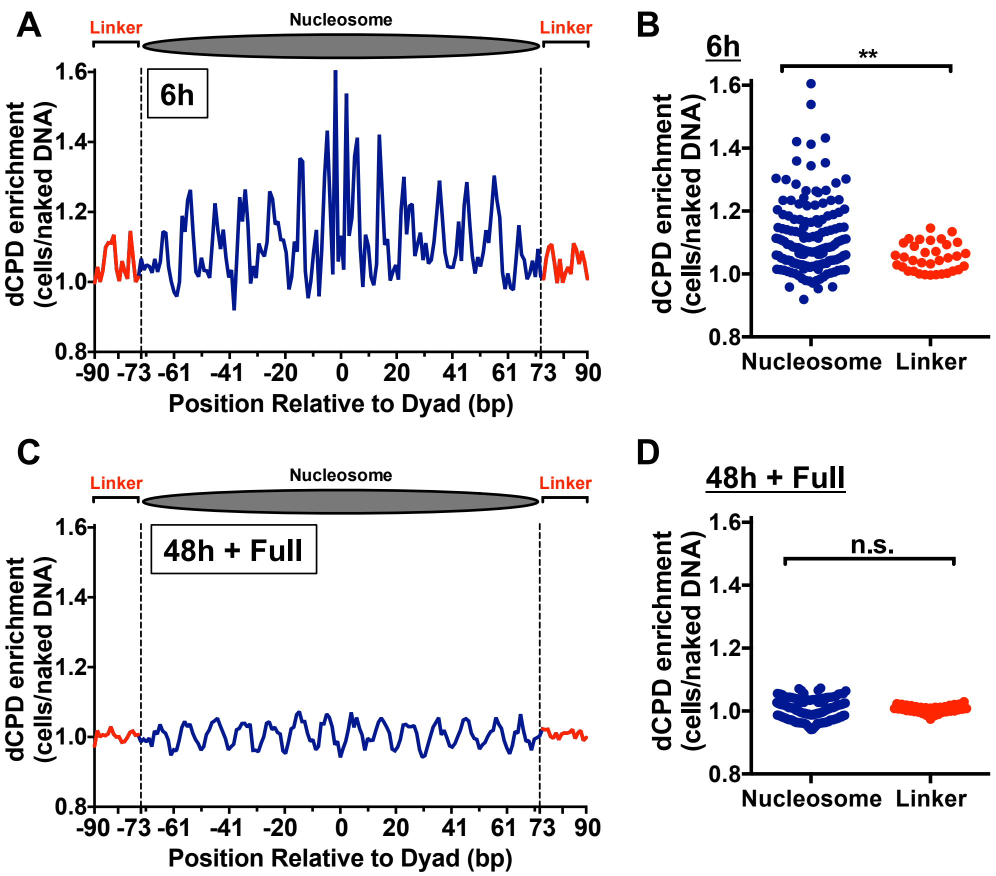

Supplemental Fig. S15. CPD deamination is elevated in nucleosome DNA relative to adjacent linker regions. (**A**) Plot of dCPD enrichment after 6h deamination in UV-irradiated cells relative to the naked DNA control in ~10,000 strongly positioned nucleosomes and adjacent linker regions (indicated in red). (**B**) Comparison of dCPD enrichment in positions within strongly positioned nucleosomes (i.e., -73 to +73 relative to dyad) relative to linker positions (i.e., -90 to -74 and +74 to +90). **P < 0.01 based on Mann Whitney *U* test. (**C-D**) Same as panels A,B, except for 48h deamination sample that was fully deaminated *in vitro*. n.s. (not significant) indicates P > 0.05, based on Mann Whitney *U* test.
