# Supplementary material for: Genome-wide maps of CPD deamination in yeast reveal the impact of DNA sequence context and nucleosome architecture on cytosine deamination rates
Source: Genome Res. 2026 Jan;36(1):183–96. doi: 10.1101/gr.280384.124 (PMC12887450; doi:10.1101/gr.280384.124)
Supplement: Supplement 17 [file Supplemental_Table_S1.pdf]

Supplemental Table S1: Yeast strains used in study.

**Strain**	**Genotype**
BY4741	MATa; *his3-1; leu2-0; met15-0; ura3-0*
yDM14	MATa; *his7-2*; *leu2-3,112; cdc13-1;* (Chr.II *lys2*Δ); (Chr.V *ura3*Δ); (Chr.V *can1*Δ); (Chr.VII *trp5*::NAT); (Chr.XV *ade2*Δ); Chr.V 36372::*lys2*::*ADE2-URA3-CAN1*
yML461	MATa; *his7-2*; *leu2-3,112; cdc13-1*; *rad14::KanMX*; (Chr.II *lys2*Δ); (Chr.V *ura3*Δ); (Chr.V *can1*Δ); (Chr.VII *trp5*::NAT); (Chr.XV *ade2*Δ); Chr.V 36372::*lys2*::*ADE2-URA3-CAN1*
